# U-Sleep’s resilience to AASM guidelines

**DOI:** 10.1038/s41746-023-00784-0

**Published:** 2023-03-06

**Authors:** Luigi Fiorillo, Giuliana Monachino, Julia van der Meer, Marco Pesce, Jan D. Warncke, Markus H. Schmidt, Claudio L. A. Bassetti, Athina Tzovara, Paolo Favaro, Francesca D. Faraci

**Affiliations:** 1 Institute of Informatics, University of Bern, Bern, Switzerland; 2 Institute of Digital Technologies for Personalized Healthcare ∣ MeDiTech, Department of Innovative Technologies, University of Applied Sciences and Arts of Southern Switzerland, Lugano, Switzerland; 3 Sleep Wake Epilepsy Center ∣ NeuroTec, Department of Neurology, Inselspital, Bern University Hospital, University of Bern, Bern, Switzerland

**Keywords:** Machine learning, Biomedical engineering

## Abstract

AASM guidelines are the result of decades of efforts aiming at standardizing sleep scoring procedure, with the final goal of sharing a worldwide common methodology. The guidelines cover several aspects from the technical/digital specifications, *e.g*., recommended EEG derivations, to detailed sleep scoring rules accordingly to age. Automated sleep scoring systems have always largely exploited the standards as fundamental guidelines. In this context, deep learning has demonstrated better performance compared to classical machine learning. Our present work shows that a deep learning-based sleep scoring algorithm may not need to fully exploit the clinical knowledge or to strictly adhere to the AASM guidelines. Specifically, we demonstrate that U-Sleep, a state-of-the-art sleep scoring algorithm, can be strong enough to solve the scoring task even using clinically non-recommended or non-conventional derivations, and with no need to exploit information about the chronological age of the subjects. We finally strengthen a well-known finding that using data from multiple data centers always results in a better performing model compared with training on a single cohort. Indeed, we show that this latter statement is still valid even by increasing the size and the heterogeneity of the single data cohort. In all our experiments we used 28528 polysomnography studies from 13 different clinical studies.

## Introduction

Since its origin in the late 1950s, polysomnography (PSG) has been at the center of sleep medicine testing with the main aim of standardizing and of simplifying the scoring procedure. A common methodology has fostered clinical research and improved sleep disorder classification and comprehension. A PSG typically involves a whole night recording of bio-signals. Brain activity, eye movements, muscle activity, body position, heart rhythm, breathing functions and other vital parameters are monitored overnight. PSG scoring is the procedure of extracting information from the recorded signals. Sleep stages, arousals, respiratory events, movements and cardiac events have to be correctly identified. Wakefulness and sleep stages, i.e., stages 1, 2, 3 and rapid eye movement (REM), can be mainly described by three bio-signals: electroencephalography (EEG), electrooculography (EOG) and electromyography (EMG). Clinical sleep scoring involves a visual analysis of overnight PSG by a human expert and may require up to two hours of tedious repetitive work. The scoring is done worldwide accordingly to official standards, *e.g*., the American Academy of Sleep Medicine (AASM) scoring manual^1^.

Artificial intelligence (AI) is a powerful technique that has the potential to simplify and accelerate the sleep scoring procedure. In literature over the last two decades, a wide variety of machine learning (ML) and deep learning (DL) based algorithms have been proposed to solve sleep scoring task^2–7^. DL based scoring algorithms have shown higher performances compared to the traditional ML approaches. Autoencoders^8^, deep neural networks (DNNs)^9^, U-Net inspired architectures^10,11^, convolutional neural networks (CNNs) and fully-CNNs^12–21^, recurrent neural networks (RNNs)^22,23^ and several combinations of them^24–32^ have been recently proposed in sleep scoring. The possibility to extract complex information from a large amount of data is one of the main reasons to apply DL techniques in PSG classification. Another significant advantage is the ability to learn features directly from raw data, by also taking into account the temporal dependency among the sleep stages.

In literature we can find many examples about how clinical guidelines have been exploited when trying to support ML and DL based algorithms. The oldest Rechtschaffen and Kales (R&K)^33^ or the updated AASM^1^ scoring manuals have been designed to cover all the aspects of the PSG: from the technical/digital specifications (*e.g*., assessment protocols, data filtering, recommended EEG derivations) to the scoring rules (*e.g*., sleep scoring rules for adults, children and infants, movement rules, respiratory rules) and the final interpretation of the results. All the sleep scoring algorithms, both ML or DL based, are trained on sleep recordings annotated by sleep physicians according to these manuals. In some of these studies the sleep recordings are pre-filtered, as indicated in the AASM guidelines, before feeding them to their scoring system. Almost all of the algorithms mentioned above are trained using recommended channel derivations and fixed length (*i.e*., 30-second) sleep epochs. However, it still remains unknown whether a DL based sleep scoring algorithm actually needs to be trained by following these guidelines. More than a decade ago, it was already highlighted that sleep is not just a global phenomenon affecting the whole brain at the same time, but that sleep patterns such as slow waves and spindle oscillations often occur out-of-phase in different brain regions^34^. Hence, it may be that DL-based scoring algorithms could retrieve the needed information from brain regions that are not necessarily the ones indicated in the AASM guidelines, reaching equally high performance. Indeed, in the growing field of mobile sleep monitoring with wearable devices, many studies are attempting to tackle the automated sleep scoring task by using unconventional channels, even not necessarily placed on the scalp, *e.g*., in-ear EEG^35–37^. Furthermore, in the AASM manual and in previous studies^38,39^, age has been addressed as one of the demographic factors that mainly change sleep characteristics (*e.g*., sleep latency, sleep cycle structure, EEG amplitude etc.). To the best of our knowledge, it has never been attempted before to incorporate this information within a sleep scoring system: it could reasonably improve its performance.

To date, all the efforts have focused on optimizing a sleep scoring algorithm in order to be ready to score any kind of subject. Data heterogeneity is one of the biggest challenges to address. A common objective among researchers is to increase the model generalizability, *i.e*., the ability of the model to make accurate predictions over different or never seen data domains. The performance of a sleep scoring algorithm on a PSG from an unseen data distribution (*e.g*., different data domains/centers) usually drastically decreases^11,30,40–42^. This drop in performance can be due to a variety of well-known reasons: high inter-scorer variability; hardware variability, *e.g*., channels/derivations; high data variability from different sleep centers, *e.g*., subject distributions with different sleep disorders. In recent studies, Phan et al. and Guillot et al.^30,40^ propose to adapt a sleep scoring architecture on a new data domain via transfer learning techniques. They demonstrate the efficiency of their approaches in addressing the variability between the source and target data domains. Perslev at al., Olesen et al. and Vallat et al.^11,41,42^ propose to train their sleep scoring architectures on tens of thousands of PSGs from different large-scale-heterogeneous cohorts. They demonstrate that using data from many different sleep centers improves the performance of their model, even on never seen data domains. In particular, Olesen et al.^41^ show that models trained on a single data domain fail to generalize on a new data domain or data center.

In our study we do several experiments to evaluate the resilience of an existing DL based algorithm against the AASM guidelines. In particular we focus on the following questions:

(i) can a sleep scoring algorithm successfully encode sleep patterns, from clinically non-recommended or non-conventional electrode derivations?
(ii) can a single sleep center large dataset contain enough heterogeneity (*i.e*., different demographic groups, different sleep disorders) to allow the algorithm to generalize on multiple data centers?
(iii) whenever we train an algorithm on a dataset with subjects with a large age range, should we exploit the information about their age, conditioning the training of the model on it?

We run all of our experiments on U-Sleep, a state-of-the-art sleep scoring architecture recently proposed by Perslev et al.^11^. U-Sleep has been chosen mainly for the following reasons: it has been evaluated on recordings from 15660 participants of 16 different clinical studies (four of them never seen by the architecture); it processes inputs of arbitrary length, from any arbitrary EEG and EOG electrode positions, from any hardware and software filtering; it predicts the sleep stages for an entire PSG recording in a single forward pass; it outputs sleep stage labels at any temporal frequency, up to the signal sampling rate, *i.e*., it can label sleep stages at shorter intervals than the standard 30-s, up to one sleep stage per each sampled time point.

In the original implementation of U-Sleep we found an extremely interesting *bug*: the data sampling procedure was not extracting the channel derivations recommended in the AASM guidelines, as stated by the authors in^11^. Instead, atypical or non-conventional channel derivations were randomly extracted. This insight triggered the above mentioned question *(i)*.

Our contributions can be summarized as follows: (1) we find that a DL sleep scoring algorithm is still able to solve the scoring task, with high performance, even when trained with clinically non-conventional channel derivations; (2) we show that a DL sleep scoring model, even if trained on a single large and heterogeneous sleep center, fails to generalize on new recordings from different data centers; (3) we show that the conditional training based on the chronological age of the subjects does not improve the performance of a DL sleep scoring architecture.

## Results

### Datasets and model experiments

We train and evaluate U-Sleep on 19578 recordings from 15,322 subjects of 12 publicly available clinical studies, as done previously^11^.

In this study we also exploit the Bern Sleep Data Base (BSDB) registry, the sleep disorder patient cohort of the Inselspital, University hospital Bern. The recordings have been collected from 2000 to 2021 at the Department of Neurology, at the University hospital Bern. Secondary usage was approved by the cantonal ethics committee (KEK-Nr. 2020-01094). The dataset consists of 8950 recordings from patients and healthy subjects aged 0–91 years. In our experiments we consider 8884 recordings, given the low signal quality of the remaining recordings. The strength of this dataset is that, unlike the ones available online, it contains patients covering the full spectrum of sleep disorders, many of whom were diagnosed with multiple sleep disorders and non-sleep related comorbidities^43^; thus providing an exceptionally heterogeneous PSG data set.

An overview of the BSDB and the open access (OA) datasets along with demographic statistics is reported in Table 1. In Supplementary notes: Datasets, we also report a detailed description of all the datasets used in this study.

**Table 1:** Datasets overview with demographic statistics.

Datasets	Recordings	Age (years)	Sex % (F/M)
^60,61^ABC (✓)	132	48.8 ± 9.8	43/57
^60,62^CCSHS (✓)	515	17.7 ± 0.4	50/50
^60,63^CFS (✓)	730	41.7 ± 20.0	55/45
^60,64,65^CHAT (✓)	1638	6.6 ± 1.4	52/48
^11^DCSM ✓	255	-	-
^60,66^HPAP (✓)	238	46.5 ± 11.9	43/57
^60,67^MESA (✓)	2056	69.4 ± 9.1	54/46
^60,68,69^MROS (✓)	3926	76.4 ± 5.5	0/100
^70,71^PHYS ✓	994	55.2 ± 14.3	33/67
^70,72^SEDF-SC ✓	153	58.8 ± 22.0	53/47
^70,72^SEDF-ST ✓	44	40.2 ± 17.7	68/32
^60,73^SHHS (✓)	8444	63.1 ± 11.2	52/48
^60,74,75^SOF (✓)	453	82.8 ± 3.1	100/0
BSDB	8884	47.9 ± 18.4	66/34

Missing values are due to study design or anonymized data. On the BSDB dataset, we compute the age and the sex values on 99.1% and on 98.6% of the whole dataset, respectively, because of missing age/sex information. Datasets directly available online are identified by ✓, while datasets that require approval from a Data Access Committee are marked by (✓). BSDB is a private dataset.

The data pre-processing and the data selection/sampling across all the datasets is implemented as described in^11^ (see subsection U-Sleep architecture). In contrast with the recommendation of the AASM manual, no filtering was applied to the EEG and the EOG signals during the pre-processing procedure. Most importantly, we found that in the original implementation of U-Sleep^11^ atypical or non-conventional channel derivations were erroneously extracted. In fact, the data extraction and the resulting sampling procedure were creating totally random derivations, see Supplementary Table 6, obviously different to those recommended in the AASM guidelines. In this study, we examine the resilience of U-Sleep with respect to the official AASM guidelines. To this aim, we extract the channel derivations following the guidelines (as was originally meant to be done in^11^), to better understand the impact of channel selection on the overall performance. Below we summarize all the experiments performed in our work on U-Sleep:

(i) We pre-train U-Sleep on all the OA datasets using both the original implementation selecting the atypical channel derivations (U-Sleep-v0), and our adaptation following AASM guidelines (U-Sleep-v1). We split each dataset in training (75%), validation (up to 10%, at most 50 subjects) and test set (up to 15%, at most 100 subjects). The split of the PSG recordings is done per-subject or per-family, *i.e*., recordings from the same subject or members of the same family appear in the same data split. In Supplementary Table 7 we summarize the data split on each OA dataset. We evaluate both U-Sleep-v0 and U-Sleep-v1 on the test set of the BSDB dataset. We also evaluate the models on the whole BSDB_(100%)_ dataset, to test on a higher number of subjects, with a higher heterogeneity of sleep disorders and a wider age range. A model pre-trained on the OA datasets and evaluated directly on the BSDB dataset is what we will refer to as direct transfer (DT) on BSDB.
(ii) We exploit the BSDB dataset to evaluate whether a DL-based scoring architecture, trained with a large and a highly heterogeneous database, is able to generalize on the OA datasets from different data centers. We split the BSDB recordings in training (75%), validation (10%) and test set (15%). We run two different experiments on U-Sleep-v1: we train the model from scratch (S) on the BSDB dataset; we fine-tune (FT) the model pre-trained in *(i)* on the BSDB dataset, by using the transfer learning approach (see subsection Transfer learning). Then, we evaluate both (S) and (FT) on the test set of all the OA datasets and the test set of the BSDB dataset.
(iii) We exploit the BSDB dataset to investigate whether U-Sleep needs to be trained by also having access to chronological age-related information. We split the BSDB dataset in seven groups, according to the age categories of the subjects^38^, resulting in *G* = 7 sub-datasets, see Supplementary notes: Age analysis. We further split the recordings of each subdataset in training (75%), validation (10% at most 50 subjects) and test set (15% at most 100 subjects). We run three different experiments on U-Sleep-v1: we fine-tune the model by using all the training sets of the seven groups (FT); we fine-tune seven independent models by using the training set of each group independently (FT-I); we fine-tune a single sandwich batch normalization model (exploiting the batch normalization layers, see subsection Conditional learning), to add the condition on the age-group-index *G* for each recording (FT-SaBN). These last two experiments are replicated considering only two age groups, *i.e*., babies/children and adults, as recommended in^1^, resulting in two additional fine-tuned model (FT-I and FT-SaBN for *G* = 2). We then evaluate all of the fine-tuned models on the independent test set of each age group.

In Supplementary Table 8 we summarize the two different data split sets, in experiment *(ii)* and experiment *(iii)*, on the BSDB dataset.

### Performance overview

(i) *Clinically non-recommended channel derivations*. In Table 2 we compare the performance of U-Sleep pre-trained on all the OA datasets, with (U-Sleep-v0) and without (U-Sleep-v1) using randomly ordered channel derivations. There is no statistically significant difference between the two differently trained architectures evaluated on the test set of the BSDB dataset (two-sided paired t-test *p* − *value* > 0.05). Most importantly, we find no difference in performance with the direct transfer also on the whole BSDB_(100%)_ dataset (two-sided paired t-test *p* − *value* > 0.05). These results clearly show how the architecture is able to generalize regardless of the channel derivations used during the training procedure, also on a never seen highly heterogeneous dataset. In Supplementary Table 9 we also compare the performance of U-Sleep-v0 and U-Sleep-v1 per sleep stage. The results suggest that there are statistically significant differences between the two differently trained architectures for each of the classes (two-sided paired t-test *p* − *value* < 0.001). U-Sleep-v0 better recognizes N1 and N3 sleep stages, at the expense of awake, N2, and REM sleep stages.

**Table 2:** *(i)* Clinically non-recommended channel derivations.

Datasets	U-Sleep-v0	U-Sleep-v1
BSDB	72.5 ± 12.2	72.5 ± 12.0
BSDB_(100%)_	72.9 ± 12.4	72.9 ± 12.4

Performance of U-Sleep-v0 and U-Sleep-v1, pre-trained on the OA datasets, and evaluated on the test set of the BSDB dataset (data split in Supplementary Table 8), and on the whole BSDB_(100%)_ dataset, *i.e*., both direct transfer (DT) on BSDB. We report the F1-score (%F1), specifically the mean value and the standard deviation (*μ* ± *σ*) computed across the recordings.
(ii) *Generalizability on different data centers with a heterogeneous dataset*. In Table 3 we report the results obtained on U-Sleep-v1 pre-trained *(i)* on the OA datasets, and evaluated on all the test sets of the OA datasets and on the test set of the BSDB dataset. We also show the results obtained on U-Sleep-v1 trained from scratch (S) on the BSDB dataset, and the results obtained on the model pre-trained in *(i)* on OA and then fine-tuned (FT) on the BSDB dataset. Unlike what we expected, both the models (S) and (FT), trained with a large and a highly heterogeneous database, are not able to generalize on the OA datasets from the different data centers. The average performance achieved on the OA with (S) and (FT) models is significantly lower compared to the performance of the model pre-trained on OA (two-sided paired t-tests *p* − *value* < 0.001). Whilst, with both (S) and (FT) we show a significant increase in performance compared to the direct transfer (DT), on the test set of the BSDB dataset (two-sided paired t-tests *p* − *value* < 0.001). We also find that the training from scratch results in significantly higher performance (two-sided paired t-test *p* − *value* < 0.001) on the BSDB dataset, compared to the performance of the fine-tuned model. No significant difference (two-sided paired t-test *p* − *value* > 0.05) occurs between (S) and (FT) evaluated on the average performance on OA datasets. The pre-training on the OA dataset is not beneficial for the model fine-tuned on the BSDB dataset. With a large number of highly heterogeneous subjects, we can directly train the model from scratch on the dataset. However, we have to mention that the main advantage of using the fine-tuned model is that it reaches same performance in less computational time, *i.e*., a fewer number of iterations (number of iterations: FT = 382 < S = 533).

**Table 3:** *(ii)* Generalizability on different data centers with a heterogeneous dataset.

Datasets	U-Sleep-v1	U-Sleep-v1 (S)	U-Sleep-v1 (FT)
ABC	73.6 ± 11.4	71.4 ± 13.9	69.0 ± 12.5
CCSHS	84.9 ± 5.1	77.3 ± 7.2	77.3 ± 6.7
CFS	76.6 ± 11.6	70.2 ± 10.8	70.9 ± 10.2
CHAT	82.1 ± 6.5	72.9 ± 8.0	68.8 ± 8.7
DCSM	79.3 ± 9.3	71.5 ± 11.2	69.3 ± 10.5
HPAP	73.8 ± 10.8	68.9 ± 11.1	67.9 ± 12.5
MESA	72.7 ± 10.8	68.5 ± 14.3	68.7 ± 11.9
MROS	71.4 ± 12.1	61.7 ± 13.7	63.9 ± 13.2
PHYS	74.2 ± 10.7	72.9 ± 11.2	73.2 ± 11.4
SEDF-SC	77.8 ± 7.9	75.8 ± 8.0	77.9 ± 7.7
SEDF-ST	77.2 ± 10.1	64.3 ± 15.4	67.5 ± 12.4
SHHS	76.9 ± 9.7	70.9 ± 9.3	73.0 ± 8.9
SOF	74.8 ± 9.8	64.6 ± 12.6	67.5 ± 11.2
avg OA	76.5 ± 10.6	69.9 ± 11.9	70.2 ± 11.1
BSDB	72.5 ± 12.0 ^(DT)^	77.6 ± 11.3	77.3 ± 11.4

Performance of U-Sleep-v1, pre-trained on the OA datasets, and evaluated on all the test sets of the OA datasets and on the test set of the BSDB dataset (data split in Supplementary Table 7 and Supplementary Table 8). We also report the performance of U-Sleep-v1 trained from scratch (S) or fine-tuned (FT) on the BSDB dataset, and evaluated on all the test sets of all the available datasets. We report the F1-score (%F1), specifically the mean value and the standard deviation (*μ* ± *σ*) computed across the recordings.
(iii) *Training conditioned by age*. In Table 4 we first show the performance of U-Sleep-v1 fine-tuned on all the training sets of the seven BSDB groups, *i.e*., single model (FT-G1). We also report the performance achieved using the training set of each group independently (FT-I) with *G* = 7 and *G* = 2 respectively (*i.e*., seven and two models), and the performance achieved using the training set of the seven/two BSDB groups conditioned (FT-SaBN) by *G* = 7 and by *G* = 2 groups respectively (*i.e*., single model). The mean and the standard deviation of the F1-score (%F1), are computed across the recordings of the test set of each of the seven BSDB age groups. Comparing both the experiments (FT-I and FT-SaBN) and types of grouping (*G* = 2 and *G* = 7) with the baseline (FT), we do not find a statistically significant increase of the performance in any of the subgroups (one-sided paired t-test *p* − *value* > 0.05). Despite the lack of significant performance differences in our age-conditioned models, REM sleep seems to be less accurately predicted for small children, if the training data set only consists of data from adults (see Supplementary Fig. 13, confusion matrix for test {*CH*} against Model 1b). This is an interesting finding since small children exhibit more REM sleep (see Supplementary Fig. 11). Visual scoring guidelines for small children differ from the guidelines for adults, with REM sleep scoring strongly relying on irregular respiration^44^. However, overall these results show that, despite the age-related differences, the DL algorithm is able to deal with different age subgroups at the same time, without needing to have access to chronological age-related information during the training procedure.

**Table 4:** *(iii)* Training conditioned by age.

Age groups	FT-G1	FT-I-G7	FT-I-G2	FT-SaBN-G7	FT-SaBN-G2
B	74.9 ± 6.8	74.1 ± 6.6 ${}^{{{{{\rm{G}}}}}_{1}}$	74.8 ± 6.2 ${}^{{{{{\rm{G}}}}}_{1}}$	72.2 ± 7.7	72.6 ± 7.7
C	75.0 ± 9.8	74.9 ± 9.2 ${}^{{{{{\rm{G}}}}}_{2}}$	75.9 ± 9.1 ${}^{{{{{\rm{G}}}}}_{1}}$	74.8 ± 8.9	75.6 ± 10.1
A	82.7 ± 13.7	80.0 ± 14.6 ${}^{{{{{\rm{G}}}}}_{3}}$	82.8 ± 13.6 ${}^{{{{{\rm{G}}}}}_{2}}$	82.3 ± 13.7	82.0 ± 14.0
YA	80.8 ± 11.5	80.6 ± 11.6 ${}^{{{{{\rm{G}}}}}_{4}}$	80.6 ± 11.6 ${}^{{{{{\rm{G}}}}}_{2}}$	80.3 ± 11.9	79.9 ± 11.9
MA	80.4 ± 7.8	79.90 ± 8.0 ${}^{{{{{\rm{G}}}}}_{5}}$	79.8 ± 8.2 ${}^{{{{{\rm{G}}}}}_{2}}$	79.6 ± 8.0	79.4 ± 8.3
E	75.7 ± 10.1	74.2 ± 10.7 ${}^{{{{{\rm{G}}}}}_{6}}$	74.9 ± 10.2 ${}^{{{{{\rm{G}}}}}_{2}}$	74.5 ± 10.6	73.9 ± 10.9
OE	75.2 ± 11.7	73.9 ± 11.0 ${}^{{{{{\rm{G}}}}}_{7}}$	74.9 ± 11.3 ${}^{{{{{\rm{G}}}}}_{2}}$	73.8 ± 11.7	74.0 ± 11.3
avg	77.9 ± 10.7	77.0 ± 10.8	77.6 ± 10.7	76.9 ± 11.0	76.8 ± 11.1

Performance of U-Sleep-v1 on a single model fine-tuned on all the training set of the seven BSDB groups (FT-G1); on seven/two models fine-tuned on the independent training set of each group with *G* = 7 (FT-I-G7) and *G* = 2 (FT-I-G2) respectively; and on a single model fine-tuned on all the training set of the seven/two BSDB groups conditioned by *G* = 7 (FT-SaBN-G7) and by *G* = 2 (FT-SaBN-G2) groups respectively. All the fine-tuned models are evaluated on the associated test set of each group (data split in Supplementary Table 8). We report the F1-score (%F1), specifically the mean value and the standard deviation (*μ* ± *σ*) computed across the recordings. B Babies (0–3 years), C Children (4–12 years), A Adolescents (13–18 years), YA Young Adults (19–39 years), MA Middle-aged adults (40–59 years), E Elderly (60–69 years), OE Old Elderly (≥ 70 years). When *G* = 2 we have the following two groups *G*_1_ = {*B* ∪ *C*}, *G*_2_ = {*A* ∪ *YA* ∪ *MA* ∪ *E* ∪ *OE*}, further details in Supplementary notes: Age analysis.

## Discussions

In this paper, we demonstrate the resilience of a DL network, when trained on a large and heterogeneous dataset. We focus on the three more significant influencing factors: channel derivation selection, multi-center heterogeneity needs, and age-conditioned fine-tuning. Channel derivations do have complementary information, and a DL-based model resulted resilient enough to be able to extract sleep patterns also from atypical and clinically non-recommended derivations. We show that the variability among different sleep data centers (*e.g*., hardware, subjective interpretation of the scoring rules, etc.) needs to be taken into account more than the variability inside one single sleep center. A large database such as the BSDB (sleep disorder patient cohort of the Inselspital, with patients covering the full spectrum of sleep disorders) does not have enough heterogeneity to strengthen the performance of the DL-based model on unseen data centers. Lastly, we show that a state-of-the-art DL network is able to deal with different age groups simultaneously, mitigating the need of adding chronological age-related information during training. In summary, what seems to be essential for the visual scoring (*e.g*., specific channel derivations, or specific scoring rules that consider also the age of the individuals) is not necessary for the DL based automatic procedure, which follows other analysis principles.

The resilience of the DL-based model to the atypical or non-conventional channel derivations is fascinating. The model still learns relevant sleep patterns while solving the scoring tasks with high state-of-the-art performance on multiple large-scale-heterogeneous data cohorts. This result proves and strengthens the feasibility to exploit alternative channels to the AASM standard ones(*e.g*., wearable applications). Although this is a remarkable finding, it would be useful to further investigate the reasons why the DL model is still able to encode clinically valid information. DL has been criticized for its non-interpretability and its black-box behavior, factors that may actually limit its implementation in sleep centers. Future works, strongly linked to the hot topic of the explainable AI, should focus on solving the following open questions: which sleep patterns/features our DL algorithms are encoding/highlighting from the typical/atypical channel derivations? How each individual channel affects the performance of the DL algorithms?

AASM scoring rules have been widely criticized over the years, for various reasons. The scoring manual has been designed to consider the sleep stages almost as discrete entities. However, it is well-known that sleep should be viewed as a continuum/gradual transition from one stage to another. A growing consensus suggests that we should reconsider the AASM scoring rules and the entire scoring procedure. Given the high variability among the individual scorers and different sleep centers, more efforts should be made by the scientific community to improve the standardization of the scoring procedure. Perhaps the introduction, even partially, of automated procedure could help.

The inter-scorer variability inevitably affects the performance of any kind of algorithm, since all algorithms are learned from the noisy variability of labels. A very relevant finding of this paper is that the heterogeneity given by data coming from different sleep data centers (*e.g*., different sleep scorers) is much more relevant than the variability coming from patients affected by different sleep disorders. These latter insights raise a research question yet to be answered: *i.e*., how could we define and quantify the heterogeneity of a sleep database? To what extent could we consider a database heterogeneous enough, to allow the algorithm to generalize across different data domains/centers?

The age-related findings drive another important observation: the DL algorithm is intrinsically encoding age-related features, which may not be categorized into discrete age-subgroups. As sleep should be considered as a continuous physiological process, the hyperspace of features associated with the respective age-subgroups should be considered continuum as well. We are forcing the algorithm to learn sleep patterns based on the chronological age of the subjects, but there are many other factors that the DL model is taking into account. Certainly, biological age has an effect on sleep characteristics. Although the DL algorithm does not need to be guided with the chronological age information during its learning procedure, it may be that with a less optimal DL-based approach (*e.g*., architecture, number of channel derivation in input) age would still be useful information to give in input.

To our knowledge, our study on the automatic sleep scoring task is the largest in terms of a number of polysomnography recordings and diversity with respect to both patient clinical pathology and age spectrum.

Considering the previous study findings and our present results, the strong resilience and the generalization capability of a DL-based architecture is undeniable. DL algorithms are now reaching better performance than the feature-based approach. DL is definitely able to extract feature representations that are extremely useful to generalize across datasets from different sleep data centers. These hidden feature representations seem to better decode the unconscious analytical evaluation process of the human scorer. To conclude, being the AASM so widely criticized, the sleep labels so noisy (*e.g*., high inter- and intra- scorer variability), and sleep so complex: could an unsupervised DL-based sleep scoring algorithm, that does not need to learn from the labels, be the solution?

## Methods

### U-Sleep architecture

U-Sleep^11^, optimized version of its predecessor U-Time^10^, is inspired by the popular U-Net architecture for image-segmentation^45–47^. Below we briefly describe U-Sleep architecture, for further details we refer the reader to^11^.

U-Sleep is a fully convolutional deep neural network. It takes as input a sequence of length *L* of 30-second epochs and outputs the predicted sleep stage for each epoch. The peculiarity of this architecture is that it defines the general function $f({{{\bf{X}}}};\theta ):{{\mathbb{R}}}^{L\cdot i\times C}\to {{\mathbb{R}}}^{L\times K}$, where *L* > 0 is any positive integer, *θ* are the learning parameters, *L* is a number of fixed-length windows with *i* sampled points each, *C* the number of PSG channels and *K* the number of sleep stages. Hence, U-Sleep takes in input any temporal section of a PSG (even the whole PSG) and output a sequence of labels for each fixed-length *i* > 0 window. Ideally *L* ⋅ *i* > 4096, because U-Sleep contains 12 pooling operations, downsampling the signal by a factor of 2. The architecture requires at least *C* = 2, one EEG and one EOG channel, sampled/resampled at 128Hz, with *K* = 5, *i.e*., *awake*, *N*1, *N*2, *N*3, *R*.

**Fig. 1:**
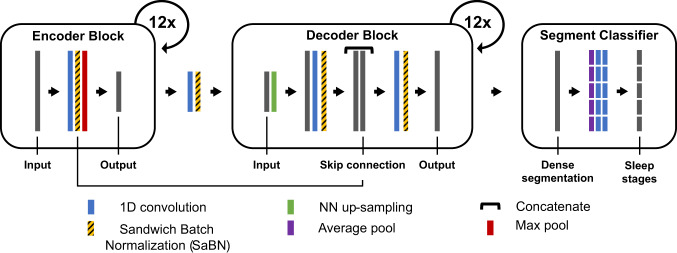
U-Sleep overall architecture. U-Sleep is a fully convolutional deep neural network. It takes as input a sequence of length *L* of 30-second sleep epochs and it outputs the predicted sleep stage for each epoch. We slightly modified the original figure (see *Figure 2: Model architecture* in^11^) reporting the additional SaBN layers exploited in the conditional learning procedure (see subsection Conditional learning). Please refer to^11^ for details on the U-Sleep model architecture and training parameters.

U-Sleep architecture consists of three learning modules as shown in Fig. 1.

- The *encoder* module is designed to extract feature maps from the input signals, each resulting in a lower temporal resolution compared to its input. It includes 12 encoder blocks. Each block consists of a 1D convolutional layer, one layer of activation function - *i.e*., exponential linear unit (ELU), a batch normalization (BN) layer and one max-pooling layer.
- The *decoder* module is designed to up-scale the feature maps to match the temporal resolution of the signals in input. We can interpret the output of the decoder as a high-frequency representation of the sleep stages at the same *f*_s_ of the input signal (*e.g*., with *f*_s_ = 128*Hz*, output one sleep stage each 1/128*Hz*). The module includes 12 decoder blocks. Each block consists of a nearest neighbor up-sampling layer (*e.g*., with a *kernel*_*size*=2, the length of the feature map in input is doubled), a 1D convolutional layer, one layer of ELU activation function and a BN layer. Then, a skip connection layer combines the up-scaled input with the output of the BN layer of the corresponding encoder block. Finally, a 1D convolution, a ELU non-linearity and a BN are applied to the stacked feature maps. The output has the same temporal resolution of the signal in input.
- The *segment classifier* module is designed to segment the high-frequency representation output of the decoder into the desired sleep stage prediction frequency. The module consist of a dense segmentation layer (*i.e*., 1d convolution layer with a hyperbolic tangent activation function), an average-pooling layer (*e.g*., with *kernel*_*size* = *stride*_*size* = 30sec*f_s_ considering the same prediction frequency of a sleep scorer) and two 1D convolutional layers (the first using an ELU activation function, and the latter using a softmax activation function). The output of the segment classifier is a *L* × *K*, where *L* is the number of segments and *K* = 5 is the number of sleep stages.

The sequence length *L*, the number of filters, the kernel and the stride sizes are specified in Fig. 1. The softmax function, together with the cross-entropy loss function, is used to train the model to output the probabilities for the five mutually exclusive classes *K* that correspond to the five sleep stages. The architecture is trained end-to-end via backpropagation, using the sequence-to-sequence learning approach. The model is trained using mini-batch Adam gradient-based optimizer^48^ with a learning rate *lr*. The training procedure runs up to a maximum number of iterations, as long as the break early stopping condition is satisfied.

Unlike^11^, we consider early stopping and data augmentation as regularization techniques. As stated in^49^ “*regularization is any modification we make to a learning algorithm that is intended to reduce its generalization error but not its training error*”. Early stopping and data augmentation do so in different ways, they both decrease the regularization error. By using the early stopping the training procedure is stopped as soon as the performance (*i.e*., F1-score) on the validation set is lower than it was in the previous iteration steps, by fixing the so called *patience* parameter. By using the data augmentation technique, the signals in input are randomly modified during training procedure to improve model generalization. Variable length of the sequences in input are replaced with Gaussian noise. For each sample in a batch, with 0.1 probability, a fraction of the sequence is replaced with $N(\mu =\hat{\mu },{\sigma }^{2}=0.01)$, where $\hat{\mu }$ is the mean of the sample’s signals. The fraction is sampled with a log-uniform distribution {*min* = 0.001; *max* = 0.33}. With a 0.1 probability at most one channel is entirely replaced by noise.

The training parameters (*e.g*., Adam-optimizer parameters beta1 and beta2, mini-batch size etc.) are all set as stated in^11^. The learning rate, the early stopping *patience* parameter and the maximum number of iterations have been changed to 10^−5^, 100, and 1000 respectively, to let U-Sleep converge faster. The architecture has several hyperparameters (*e.g*., number of layers, number/sizes of filters, regularization parameters, training parameters, etc.) which could be optimized to tune its performance on any dataset. We decide to not systematically tune all these parameters, as this is out of our scope, but to fix them for all the experiments, as done in the original network.

#### Data pre-processing

The signals are resampled to 128 Hz and rescaled (per channel and per-subject), so that, for each channel, the EEG signal has median 0 and inter quartile range (IRQ) 1. The values with an absolute deviation from the median above 20*IQR are clipped. The signals outside the range of the scored hypnogram are trimmed. The recordings scored according to Rechtschaffen and Kales rules results in six scoring classes, *i.e*., awake, N1, N2, N3, N4, and REM. In order to use the AASM standard, we merge the N3 and N4 stages into a single stage N3. The loss function for stages as MOVEMENT and UNKNOWN is masked during the training procedure.

#### Data sampling

U-Sleep is trained using mini-batch Adam gradient-based optimizer. Each element in the batch is a sequence/segment of *L* = 35 EEG and EOG 30-second signals/epochs from a single subject. Each sequence/element is sampled from the training data as follows. (1) dataset sampling: one dataset is selected randomly. The probability that a dataset *D* is selected is given by *P*(*D*) = *αP*_1_(*D*) + (1 − *α*)*P*_2_(*D*), where *P*_1_(*D*) is the probability that a dataset is sampled with a uniform distribution 1/*ND*, where *ND* is the number of available datasets, and *P*_2_(*D*) is the probability of sampling a dataset according to its size. The parameter *α* is set to 0.5 to equally weight *P*_1_(*D*) and *P*_2_(*D*); (2) subject sampling: a recording *SD* is uniformly sampled from *D*; (3) channel sampling: one EEG and one EOG are uniformly sampled from the available combinations of channels in *SD* (*e.g*., if 2 EEG and 2 EOG channels are available, four combinations are possible); (4) segment sampling: a segment of EEG signal and a segment of EOG signal, both of length *L* = 35, are selected as follows: first a class from *W*, *N*1, *N*2, *N*3, *R* is uniformly sampled, then a 30-second epoch scored with the sampled class is selected randomly from the whole night recording, the chosen epoch is shifted into a random position of the segment of length *L* and finally the sequence is extracted.

### Transfer learning

We define transfer learning as in the following clear and simple statements:

"*Transfer learning and domain adaptation refer to the situation where what has been learned in one setting* (*e.g*., *distribution* *P*_1_) *is exploited to improve generalization in another setting (say, distribution* *P*_2_)”^49^;

"*Given a source domain* *D*_*S*_ *and learning task* *T*_*S*_, *a target domain* *D*_*T*_ *and learning task* *T*_*T*_, *transfer learning aims to help improve the learning of the target predictive function* *f*_*T*_( ⋅ ) in *D*_*T*_ *using the knowledge in* *D*_*S*_ *and* *T*_*S*_, *where* *D*_*S*_ ≠ *D*_*T*_ *and* *T*_*S*_ ≠ *T*_*T*_”^50^.

In our study the source and the target tasks are the same, i.e. *T*_S_ ≡ *T*_T_. The task is always to perform sleep staging with the same set of sleep classes/stages. We want to transfer the knowledge about the previously learned sleep recordings (*e.g*., different hardware, different subject distributions with different sleep disorders) and the knowledge about the sleep scoring-rules (*i.e*., inter-scorer variability in the different data centers). The process generally involves overwriting a knowledge from a small-sized database to a previous big-sized knowledge (result of a long training process). One big concern is to avoid ending up in what the data scientists call catastrophic forgetting: “*Also known as catastrophic interference, it is the tendency of an artificial neural network to completely and abruptly forget previously learned information upon learning new information*” as defined in^51^. Even if it is conceptually easy to understand, avoiding its occurrence is not trivial. To partially bypass this phenomena we fine-tune the architecture on the target domain using a smaller learning rate.

In our experiments we first pre-train the architecture on the data-source domain *S* (*e.g*., a set of different domains/databases $\{{S}_{{{{{\rm{DB}}}}}_{1}},{S}_{{{{{\rm{DB}}}}}_{2}},...,{S}_{{{{{\rm{DB}}}}}_{{{{\rm{n}}}}}}\}$), then we fine-tune the model on the data-target domain *T*. Formally, we first minimize the loss function *L*_S_, resulting in the learned parameters *θ*:

1 $$argmi{n}_{\theta }=\mathop{\sum }\limits_{({{{\bf{x}}}},{{{\bf{y}}}})\in {D}_{{{{\rm{S}}}}}}^{}{L}_{{{{\rm{S}}}}}({{{\bf{x}}}},P({{{\bf{y}}}}\parallel {{{\bf{x}}}}),{P}_{\theta }({{{\bf{y}}}},{{{\bf{x}}}}))$$

The parameters *θ* of the pre-trained model are used as the starting point on the data-target domain *T*. To transfer the learning on the new domain *T*, we fine-tune all the pre-trained parameters ${\theta }^{{\prime} }=\theta$ (*i.e*., the entire network is further trained on the new data domain *T*):

2 $$argmi{n}_{{\theta }^{{\prime} } = \theta }=\mathop{\sum }\limits_{({{{\bf{x}}}},{{{\bf{y}}}})\in {D}_{{{{\rm{T}}}}}}^{}{L}_{{{{\rm{T}}}}}({{{\bf{x}}}},P({{{\bf{y}}}}\parallel {{{\bf{x}}}}),{P}_{\theta }({{{\bf{y}}}},{{{\bf{x}}}}))$$

### Conditional learning

Basically all the sleep scoring architectures learn in a conditional way. The aim is to maximize the conditional probability distributions *P*(**Y**∣**X**), where **X** are the sequences of the biosignals in input and **Y** are the corresponding ground-truth labels. For each epoch **x**_*t*_ in input the models aim to maximize the conditional probability distribution *P*(*y*_*t*_∣**x**_*t*_), where *y*_*t*_ is the *t* − *th* one-hot encoded vector of the ground-truth label. Hence, the model is trained to minimize the prediction error conditioned only by the knowledge of **X**. We know that the sleep data **X** often come from different sources or data domains. Even in the same cohort, subjects with different demographics and sleep disorders may occur, resulting in significant shifts in their sleep data **X** distributions. Imagine to have in the same data cohort *G* different groups of subjects $\left\{{g}_{1},{g}_{2},...,{g}_{{{{\rm{G}}}}}\right\}$, with ${g}_{1}=\left\{healthy\right\}$, *g*_2_ = {*sleep*_*apnea*} and so on. This additional information about the group (*i.e*., the sleep disorder group *g*_i_) to which the subject belongs can be given in input to the model. So, we can either train *G* fully separated models, each maximizing *G* different *P*(**Y**∣**X**) functions, or either train a single model maximizing the conditional probability distributions *P*(**Y**∣**X**, *g*_i_). The latter - *i.e*., train the joint model with the additional condition *g*_i_ - is the smartest approach; the tasks are similar enough to benefit from sharing the parameters and the extracted features.

We decide to exploit the BN layers to insert the additional knowledge in the training of our model. In literature different normalization variants have been proposed by modulating the parameters of the vanilla BN layer^52–56^. We decide to exploit the sandwich batch normalization (SaBN) approach recently proposed in^57^.

The vanilla BN^58^ normalizes the samples in a mini-batch in input by using the mean *μ* and the standard deviation *σ*, and then re-scales them with the *γ* and *β* parameters. So, given the feature in input $f\in {{\mathbb{R}}}^{B\times C\times H\times W}$, where *B* is the batch size, *C* is the number of channels and *H* and *W* are the height and width respectively, the vanilla BN computes:

3 $$h=\gamma (\frac{f-\mu (f)}{\sigma (f)})+\beta$$

where *μ*(*f*) and *σ*(*f*) are the mean and variance running estimates (batch statistics, *i.e*., moving mean and moving variance) computed on *f* along (*N*, *H*, *W*) dimensions; *γ* and *β* are the re-scaling learnable parameters of the BN affine layer with shape *C*. Clearly, the vanilla BN has only a single re-scaling transform, indirectly assuming all features coming from a single data distribution. In^55^, to tackle the data heterogeneity issue (*i.e*., images from different data domains/distributions), they propose the Categorical Conditional BN (CCBN), so boosting the quality of the generated images. The CCBN layer computes the following operation:

4 $$h={\gamma }_{{{{\rm{g}}}}}(\frac{f-\mu (f)}{\sigma (f)})+{\beta }_{{{{\rm{g}}}}}\qquad g=1,...,G$$

where *γ*_g_ and *β*_g_ are the re-scaling learnable parameters of each *g* − *th* affine layer, where *g* corresponds to the domain index associated to the input. The parameters of each affine layer are learned to capture the domain/distribution-specific information. In^57^, instead, they propose the SaBN layer, an improved variant of the CCBN. They claim that different individual affine layers might cause an imbalanced learning for the different domains/distributions. They factorize the BN affine layer into one shared “sandwich” BN layer cascaded by a set of independent BN affine layers, computed as follows:

5 $$h={\gamma }_{{{{\rm{g}}}}}({\gamma }_{{{{\rm{sa}}}}}(\frac{f-\mu (f)}{\sigma (f)})+{\beta }_{{{{\rm{sa}}}}})+{\beta }_{{{{\rm{g}}}}}\qquad i=1,...,G$$

where *γ*_sa_ and *β*_sa_ are the re-scaling learnable parameters of the “sandwich” shared affine BN layer, while, as above, *γ*_g_ and *β*_g_ are the re-scaling learnable parameters of each *g* − *th* affine layer, conditioned on the categorical input *g*. The SaBN enable the conditional fine-tuning of a pre-trained U-Sleep architecture, conditioned by the categorical index in input *g*.

### Evaluation

In all our experiments we evaluate U-Sleep as stated in^11^. The model scores the full PSG, without considering the predicted class on a segment with a label different from the five sleep stages (*e.g*., segment labeled as ’UNKNOWN’ or as ’MOVEMENT’). The final prediction is the results of all the possible combinations of the available EEG and EOG channels for each PSG. Hence, we use the majority vote, *i.e*., the ensemble of predictions given by the multiple combination of channels in input.

The unweighted F1-score metric^59^ is computed on all the testing sets to evaluate the performance of the model on all the experiments. We compute the F1-score for all the five classes, we then combine them by calculating the unweighted mean. Note that the unweighted F1-scores reduce the absolute scores due to lower performance on less abundant classes such as sleep stage N1. For this reason, we also report in Supplementary Table 10, Supplementary Table 11, and Supplementary Table 12 the results achieved in terms of weighted F1-score - *i.e*., the metric is weighted by the number of true instances for each label, so as to consider the high imbalance between the sleep stages. In that case, the absolute scores significantly increases on all the experiments. In Supplementary Table 10, Supplementary Table 11, and Supplementary Table 12 we also report the Cohen’s kappa metric, given its valuable property of correcting the chance of agreement between the automatic sleep scoring algorithm, *i.e*., overall predicted sleep stages, and the ground truth, *i.e*., the sleep labels given by the physicians.

* The Bern Sleep Data Base BSDB registry usage was ethically approved in the framework of the E12034 - SPAS (Sleep Physician Assistant System) Eurostar-Horizon 2020 program (Kantonale Ethikkommission Bern, 2020-01094).

### Reporting summary

Further information on research design is available in the Nature Research Reporting Summary linked to this article.

### Supplementary information

Supplemental Information

REPORTING SUMMARY

## Data Availability

The Bern Sleep Data Base BSDB registry, the sleep disorder patient cohort of the Inselspital, University Hospital Bern, is not publicly available. The BSDB data are available on request from the corresponding author L.F. (legal conditions ensuring data privacy will be defined in a “data transfer agreement document”, together with a description of the analysis project). All other datasets are in principle publicly available, most datasets require the user to complete a data request form. The researchers and the use-case scenario need to be eligible for a given dataset. In Table 1 we specify which datasets require approval from a Data Access Committee and which are directly available online.

## Code availability

The code we used in our study is based on what was previously developed in^11^, publicly available on GitHub at https://github.com/perslev/U-Time. All our experiments were carried out using the following branch https://github.com/perslev/U-Time/tree/usleep-paper-version. As a result of important feedback received from the whole community, but especially thanks to our important feedback related to the use of atypical and clinically non-recommended derivations, the authors provide the bugfixed code in https://github.com/perslev/U-Time/tree/usleep-paper-version-branch-bugfixes.
