# Supplementary material for: U-Sleep’s resilience to AASM guidelines
Source: NPJ Digit Med. 2023 Mar 6;6:33. doi: 10.1038/s41746-023-00784-0 (PMC9988983; doi:10.1038/s41746-023-00784-0)
Supplement: Supplementary file 1 — Supplemental Information [file 41746_2023_784_MOESM1_ESM.pdf]

U-Sleep's resilience to AASM guidelines

Luigi Fiorillo[1,2*†], Giuliana Monachino[1,2†], Julia van der Meer[3], Marco Pesce[3], Jan D. Warncke[3], Markus H. Schmidt[3], Claudio L.A. Bassetti[3], Athina Tzovara[1,3], Paolo Favaro[1] and Francesca D. Faraci[2]

[1*]Institute of Informatics, University of Bern, Neubrückstrasse 10, Bern, 3012, Switzerland.

[2]Institute of Digital Technologies for Personalized Healthcare | MeDiTech, Department of Innovative Technologies, University of Applied Sciences and Arts of Southern Switzerland, Via la Santa 1, Lugano, 6962, Switzerland.

[3]Sleep Wake Epilepsy Center | NeuroTec, Department of Neurology, Inselspital, Bern University Hospital, University of Bern, Freiburgstrasse 16, Bern, 3010, Switzerland.

*Corresponding author(s). E-mail(s): luigi.fiorillo@supsi.ch;
†These authors contributed equally to this work.

SUPPLEMENTARY MATERIAL

Supplementary notes

Dataset

We report a detailed description of all the datasets used in our experiments.

BSDB. The Bern Sleep Data Base consists of 8950 recordings from 7985 subjects. A small percentage of the subjects is healthy (below 1%). The rest of the subjects are patients with a single or multiple sleep disorders or with an uncertain diagnosis. The most common class of sleep disorders is sleep related breathing disorders, followed by central disorders of hypersomnolence, insomnia, parasomnias and sleep related movement disorders. A smaller percentage of patients with circadian rhythm sleep-wake disorders and isolated symptoms and normal variants is also present. EEG signals (F4-M1, F3-M2, C4-M1, C3-M2, O2-M1, O1-M2) and EOG signals (E2-M1, E1-M2) are considered in our experiments. The signals are recorded at 200Hz. The recordings are manually scored by sleep experts according to the AASM rules. Given the different scoring rules for infants ($\leq$ 2 months) [1], it is important to specify, in the context of the following age analysis, that in the BSDB dataset there were no babies younger than two months.

ABC. The Apnea, Bariatric surgery, and CPAP database consists of 132 recordings from 49 patients with severe obstructive sleep apnea and morbid obesity (BMI from 35 to 45) [2, 3]. EEG signals (F4-M1, F3-M2, C4-M1, C3-M2, O2-M1, O1-M2) and EOG signals (E2-M1, E1-M2) are considered in our experiments. The signals are recorded at 256Hz, and are hardware low-pass filtered at 105Hz and high-pass filtered at 0.16Hz. The recordings are manually scored by sleep experts according to the AASM rules. For more information we refer to https://doi.org/10.25822/nx52-bc11 and https://clinicaltrials.gov/ct2/show/NCT01187771.

CCSHS. The Cleveland Childrend's Sleep and Health Study consists of children and adolescents recordings. In our experiments we consider 515 recordings from adolescents aged 16-19 years. A small percentage of the subjects suffers from sleep related movement disorders. The recordings are collected in three different hospitals around Cleveland, Ohio, US [2, 4]. EEG signals (C4-A1, C3-A2) and EOG signals (ROC-A1, LOC-A2) are considered in our experiments. The signals are recorded at 128Hz, and hardware high-pass filtered at 0.15Hz. The recordings are manually scored by sleep experts according to the AASM rules. For more information we refer to https://doi.org/10.25822/cg2n-4y91.

CFS. The Cleveland Family Study is a family-based study on sleep apnea disordered subjects. The database consists of 2284 subjects from 361 families

[2, 5]. We consider recordings of 730 subjects from 144 families (whence full whole-night PSG were available). For this specific database, the data split (train/val/test set) is done by considering subjects and family belonging (*i.e.*, all the family members appear in the same data split). More than half of the subjects are affected by sleep apnea disorder. EEG signals (C4-A1, C3-A2) and EOG signals (ROC-A1, LOC-A2) are considered in our experiments. The signals are recorded at 128Hz, and hardware low-pass filtered and high-pass filtered at 105Hz and 0.16 Hz respectively. The recordings are manually scored by sleep experts according to the AASM rules. For more information we refer to https://doi.org/10.25822/jmyx-mz90.

CHAT. The Childhood Adenotonsillectomy Trial database consists of 1638 recordings (452 baseline, 407 follow-up and 779 control) from 1232 children post-adenotonsillectomy-surgery aged 5-10 years with mild to moderate obstructive sleep apnea. The recordings are collected in six different sleep centers in Massachusetts, Missouri, New York, Ohio and Pennsylvania [2, 6, 7]. EEG signals (F4-M1, F3-M2, C4-M1, C3-M2, O2-M1, O1-M2, T4-M1, T3-M2) and EOG signals (E2-M1, E1-M2) are considered in our experiments. The signals are recorded at 200Hz (or higher in other sleep centers), and different hardware filtering given the different acquisition systems. One recording is excluded - EOG missing. The recordings are manually scored by sleep experts according to the AASM rules. For more information we refer to https://doi.org/10.25822/d68d-8g03 and https://clinicaltrials.gov/ct2/show/NCT00560859.

DCSM. The Danish Centre for Sleep Medicine database consists of 255 recordings from patients with potential and non-specific sleep related disorders [8]. No demographic information is available for the database. EEG signals (F4-M1, F3-M2, C4-M1, C3-M2, O2-M1, O1-M2, T4-M1, T3-M2) and EOG signals (E2-M1, E1-M2) are considered in our experiments. The signals are recorded at 256Hz, and band-pass filtered between 0.3Hz and 70Hz. The recordings are manually scored by sleep experts according to the AASM rules. For more information we refer to https://sid.erda.dk/wsgi-bin/ls.py?share_id=fUH3xbOXv8.

HPAP. The Home Positive Airway Pressure database consists of 373 recordings (238 considered in our experiments) from obstructive sleep apnea patients aged over 18 years. The recordings are collected in seven different US sleep centers [2, 9]. EEG signals (F4-M1, F3-M2, C4-M1, C3-M2, O2-M1, O1-M2, T4-M1, T3-M2) and EOG signals (E2-M1, E1-M2) are considered in our experiments. The signals are recorded at 200Hz, no filtering applied. Nine recordings are excluded - EOG and/or reference channels missing. The recordings are manually scored by sleep experts according to the AASM rules. For more information we refer to https://doi.org/10.25822/xmwv-yz91 and https://clinicaltrials.gov/ct2/show/NCT00642486.

MESA. The Multi-Ethnic Study of Atherosclerosis consists of 2237 recordings (2056 considered in our experiments) from a cohort of black, white, Hispanic and Chinese-American subjects aged 45-84 years [2, 10]. About 15.0% of individuals have severe SDB, 30.9% short sleep duration, 6.5% poor sleep quality and 13.9% daytime sleepiness. EEG signals (Fz-Cz, C4-M1, Cz-Oz) and EOG signals (E2-Fpz, E1-Fpz) are considered in our experiments. The signals are recorded at 256Hz, and hardware low-pass filtered at 100Hz. The recordings are manually scored by sleep experts according to the AASM rules. For more information we refer to https://doi.org/10.25822/n7hq-c406.

MROS. The database is a subset of the larger study Osteoporotic Fractures in Men, involving 5994 community-dwelling men aged over 65 years [2, 11, 12]. In our experiments we consider 3926 recordings (2900 from visit 1 and 1026 from visit 2) from 2903 subjects, which underwent in-home overnight PSG. Most of the subjects are sleep breathing disorders patients. EEG signals (C4-A1, C3-A2) and EOG signals (ROC-A1, LOC-A2) are considered in our experiments. The signals are recorded at 256Hz, and hardware high-pass filtered at 0.15Hz. Seven recordings are excluded - EOG channels and/or sleep stage annotation files missing. The recordings are manually scored by sleep experts according to the AASM rules. For more information we refer to https://doi.org/10.25822/kc27-0425.

PHYS. The database from the 1028 PhysioNet/CniC Challenge consists of 1985 recordings (994 labelled considered in our experiments) from patients with potential sleep disorders [13, 14]. EEG signals (F4-M1, F3-M2, C4-M1, C3-M2, O2-M1, O1-M2) and one EOG signal (E1-M2) are considered in our experiments. The signals are recorded at 200Hz. The recordings are manually scored by sleep experts according to the AASM rules. For more information we refer to https://physionet.org/content/challenge-2018/1.0.0/.

SEDF-SC & SEDF-ST. The Sleep-EDF Expanded database consists of 197 recordings from two subset studies. The Sleep-EDF Sleep Cassette consists of 153 recordings from 78 healthy subjects aged 25-101 years. The Sleep-EDF Sleep Telemetry consists of 44 recordings from 22 healthy subjects with mild difficulty falling asleep (two recordings collected for each subject, *i.e.*, one after temazepam intake and one after placebo intake) [13, 15]. EEG signals (Fpz-Cz, Pz-Oz) and one EOG signal (ROC-LOC) are considered in our experiments. The signals are recorded at 100Hz. The recordings are manually scored by sleep experts according to the Rechtschaffen and Kales scoring rules, and re-aligned to the AASM rules. For more information we refer to https://doi.org/10.13026/C2C30J.

SHHS. The Sleep Heart Health Study consists of 8444 recordings (5793 from visit 1 and 2651 from visit 2) from 5797 subjects aged over 40 years [2, 16]. Most of the subjects suffer from OSA or other SDB. EEG signals

(C4-A1, C3-A2) and EOG signals (ROC-A1, LOC-A2) are considered in our experiments. The EEG and EOG signals are recorded at 125Hz and 50Hz respectively, and hardware high-pass filtered at 0.15Hz. The recordings are manually scored by sleep experts according to the Rechtschaffen and Kales scoring rules, and re-aligned to the AASM rules. For more information we refer to https://clinicaltrials.gov/ct2/show/NCT00005275 and https://doi.org/10.25822/ghy8-ks59.

SOF. The database is a subset of the larger study Osteoporotic Fractures. In our experiments we consider 453 recordings (from visit 8), which underwent in-home overnight PSG [2, 17, 18]. EEG signals (C4-A1, C3-A2) and EOG signals (ROC-A1, LOC-A2) are considered in our experiments. The EEG and EOG signals are recorded at 128Hz, and hardware high-pass filtered at 0.15Hz. The recordings are manually scored by sleep experts according to the Rechtschaffen and Kales scoring rules, and re-aligned to the AASM rules. For more information we refer to https://doi.org/10.25822/e1cf-rx65.

Age analysis

(G=7) Age groups by [19]

Inspired by the meta-analysis of quantitative sleep parameters from childhood to old age reported in [19], we decide to study, and then run all our experiments, on the following seven age groups: Babies (B; 0-3 years), Children (C; 4-12 years), Adolescents (A; 13-18 years), Young Adults (YA; 19-39 years), Middle Aged Adults (MA; 40-59 years), Elderly (E; 60-69 years), Old Elderly (OE; $\geq$70 years). Unlike in [19], we also consider the additional group of Babies, uncovered in their study. A total of 8869 PSGs from the BSDB dataset, the ones for which the age was available, are considered in this analysis. In Supplementary Figure 1 and in Supplementary Table 1, respectively, we show the age distribution of the dataset in the seven age groups, and we report the number of recordings, the age range, the age mean and standard deviation ($\mu \pm \sigma$) and the male/female percentage (M/F) for each group. For each PSG of the dataset (except for 6 recordings containing only awake sleep stages) we compute the following ten sleep parameters: Total Sleep Time (TST), Sleep Period Time (SPT), Wake After Sleep Onset (WASO), Sleep Latency (SL), Sleep Efficiency (SE), Percentage of N1 stage (pN1), Percentage of N2 stage (pN2), Percentage of N3 stage (pN3), Percentage of REM stage (pREM) and Number of stage shifts per hour (n_shift). In Supplementary Table 2 we report the mean and standard deviation ($\mu \pm \sigma$) of each sleep parameter for each age group. In Supplementary Figures 3-12 we also report the boxplots computed on each sleep parameter and for each age group. In some of these plots emerge the continuous positive/negative trend on the specific sleep parameter from babies to old elderly subjects.

(G=2) Age groups by AASM [1]

With sleep-specific age groups we refer to those suggested by the AASM scoring manual [1]. Indeed, it provides two sets of rules for visual sleep scoring, *i.e.*, babies/children and adults.
The age boundary between the two groups is not well defined; in particular, it is not clear which group the subjects in the age range between 13 and 18 (adolescents) belong to. Therefore we started considering two groups plus the adolescents' group: Babies/Children ($CH = \{B + C\}$; 0-12 years), Adolescents (A; 13-18 years), Adults ($AD = \{YA + MA + E + OE\}$; $\geq$ 19 years).

In Supplementary Figure 2 and in Supplementary Table 3, respectively, we show the age distribution of 8869 recordings of the BSDB dataset in the three age groups, and we report the number of recordings, the age range, the age mean and standard deviation ($\mu \pm \sigma$) and the male/female percentage (M/F) for each age group. We exploit U-Sleep to evaluate if the PSGs of the Adolescents were closer to the Babies/Children's recordings or to the Adults' recordings. We run two different experiments on U-Sleep-v1: in experiment_1 we merge the recordings from the Adolescents with the Babies/Children; in experiment_2 we merge the recordings from Adolescents with the Adults. In both the experiments (1, 2), we fine-tune two different models (a, b), resulting in four independently trained models: (1a) fine-tuning on $G_{\text{1a}} = \{CH + A\}$; (1b) fine-tuning on $G_{\text{1b}} = \{AD\}$; (2a) fine-tuning on $G_{\text{2a}} = \{CH\}$; (2b) fine-tuning on $G_{\text{2b}} = \{A + AD\}$. For each experiment, we test both the models (a) and (b) on the test set of the three groups $\{CH, A, AD\}$. In Supplementary Table 4 we report the macro F1-score (%F1), specifically the mean value and the standard deviation ($\mu \pm \sigma$), computed across the recordings. In bold we indicate the best performance achieved on each test set. We compare with a two-sided paired t-test the performance of the four models tested on the Adolescents. The model (2b), fine-tuned on $\{A + AD\}$, performs significantly better (two-sided paired t-test $p - value < 0.05$) on the Adolescents than the model (1a), fine-tuned on $\{CH + A\}$, suggesting that Adolescents tend to be similar similar to Adults. This is confirmed by the fact that also the model (1b), fine-tuned on $\{AD\}$, performs better (two-sided paired t-test $p - value <$ 0.05) on the Adolescents than the model (1a), even without Adolescents' recordings in the training set. The models (1b) and (2b), fine-tuned on Adults without or with Adolescents, reach the same performance (two-sided paired t-test $p - value > 0.05$), hence confirming again the statement above. Following this indication we run all the age conditioning analysis on these two sleep-related age groups: $G_1 = \{B + C\}$ and $G_2 = \{A + YA + MA + E + OE\}$. For both $\{CH\}$ and $\{AD\}$ we obtain the same performance (two-sided paired t-test $p - value > 0.05$) with the two models fine-tuned on the group itself, with or without Adolescents. However, we reach significantly lower performance (two-sided paired t-test $p - value < 0.01$) with the other two models fine-tuned on the complementary group. This latter statement strengthens the basic assumption that babies/children and adults are two clearly different

groups for the deep learning scoring algorithm.

In Supplementary Figure 13 we report the confusion matrix for each of the five models (0, 1a, 1b, 2a, 2b) and each of the three test sets $\{CH, A, AD\}$. With model (0) we refer to the model fine-tuned on the whole training set, regardless of the subjects' age.

Noisy labels, uncertainty and query procedure

The visual scoring is a highly subjective procedure, indeed the AASM rules leave space for subjective interpretation. In the last decades, [20–23] have reported high inter-scorer variability, *i.e.*, the agreement between different scorers is of about 70%-80%. The labels we are using to train our sleep scoring algorithms are commonly annotated by a single scorer. Hence, we are actually transferring the scorer's subjectivity in our models. The labels can then be considered noisy by nature. This noise may generate uncertainty during the training procedure. In our study we also exploit the label smoothing regularization technique to introduce additional noise on top of our labels, to better evaluate the uncertain predictions given from our model. In [24, 25] it has been shown that label smoothing helps improving robustness when learning with noisy labels.

The predicted sleep stage for each fixed-length $i > 0$ window comes with a probability value $\hat{p}$. As stated in [26], the probability value associated with the predicted sleep stage should mirror its ground truth correctness likelihood. When this happens the model is well calibrated. In [26] we also showed label smoothing [27] to be a suitable technique to improve the calibration of the model. In this study, we also train U-Sleep with the label smoothing techniques, to add some noise on the labels, to better calibrate the model and to evaluate its impact on our uncertainty estimate procedure. In a standard training of a neural network, the cross-entropy loss is minimized using the hard targets y_{k} (*i.e.*, hot encoded targets, '1' for the correct class and '0' for the other). When the model is trained with the label smoothing technique, the hard targets are weighted with the uniform distribution $1/K$ (equation (1)), and the cross-entropy loss is minimized using the weighted mixture of the targets (equation (2)).

$$ {y_{\mathrm{k}}}^{LS_{\mathrm{U}}} = y_{\mathrm{k}} \cdot (1-\alpha) + \alpha/K \tag{1} $$

$$ H(\mathbf{y}, \mathbf{p}) = \sum_{k=1}^{K} -{y_{\mathrm{k}}}^{LS_{\mathrm{U}}} \cdot \log(\hat{p}_{\mathrm{k}}) \tag{2} $$

where α is the smoothing parameter, K is the total number of classes, ${y_{\mathrm{k}}}^{LS_{\mathrm{U}}}$ the targets smoothed with the uniform distribution, and $\hat{p}_{\mathrm{k}}$ the softmax output probabilities. We fix the α smoothing parameter equal to 0.1.

We exploit the ensemble of the M different predictions (*i.e.*, one prediction for each combination of channel in input) and the query procedure introduced in [26] to estimate the model uncertainty, and consequently the uncertain predictions. We can compute the mean $\mu_{\mathrm{i,k}}$ (equation (3)) and the variance ${\sigma^2}_{\mathrm{i,k}}$ (equation (4)) of the M predictions for each sleep stage k :

$$ \mu_{\mathrm{i,k}} = \frac{\sum_{m=1}^{M} \hat{p}_{\mathrm{m,i,k}}}{M} \tag{3} $$

$$\sigma^2{}_{\mathrm{i,k}} = \frac{\sum_{m=1}^{M} (\hat{p}_{\mathrm{m,i,k}} - \mu_{\mathrm{i,k}})^2}{M} \quad (4)$$

where $\hat{p}_{\mathrm{m,i,k}}$ is the output probability for the sleep stage k of the m-th prediction for the $i-th$ 30-second epoch. The final prediction $\hat{y}_{\mathrm{i}}$ of the model will be given by $max(\mu_{\mathrm{i}})$, which we will refer to as μ_{max}, along with the assigned variance value $\sigma^2{}_{\mu\mathrm{max}}$.
As proposed in [26], the mean μ_{max} and the variance $\sigma^2{}_{\mu\mathrm{max}}$ can be used as indicators of the model uncertainty. High μ_{max} and low $\sigma^2{}_{\mu\mathrm{max}}$ indicate that the model is confident about its prediction, *i.e.*, low degree of uncertainty. In this study, we use only the μ_{max} query procedure introduced in [26], *i.e.*, on each subject we select a fixed percentage of epochs with the lowest μ_{max} value. It has been shown to be more efficient compared to the query procedure via $\sigma^2{}_{\mu\mathrm{max}}$. The query procedure requires the selection of a threshold q% on the distribution of the mean values. The selection criterion of the threshold value q% is based on a reasonable percentage of epochs to be re-sent to the physician for a secondary review. In our study we fix q% equal to 5%, as done in [26].

We find that training U-Sleep-v1 on the OA datasets with the label smoothing technique results in a significant decrease in performance compared to the baseline pre-trained in *(ii)* without label smoothing (two-sided paired t-test $p-value < 0.001$). Nonetheless, by adding some noise on the label during the training, we are able to select a significantly higher number of misclassified epochs among the selected ones (two-sided paired t-test $p-value < 0.001$). We thus better detect the uncertain predictions, see Supplementary Table 5.

List of Supplementary Figures

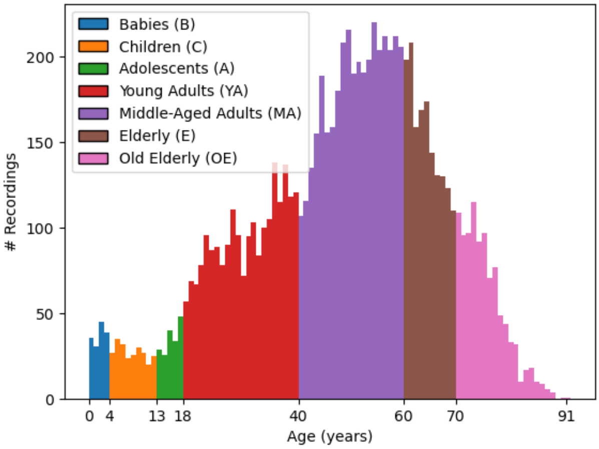

Supplementary Figure 1 Age distribution of the BSDB dataset (n=8869) in the seven age groups by [19]. B: Babies (0-3 years); C: Children (4-12 y); A: Adolescents (13-18 y); YA: Young Adults (19-39 y); MA: Middle Aged Adults (40-59 y); E: Elderly (60-69 y); OE: Old Elderly ($\geq$ 70 y).

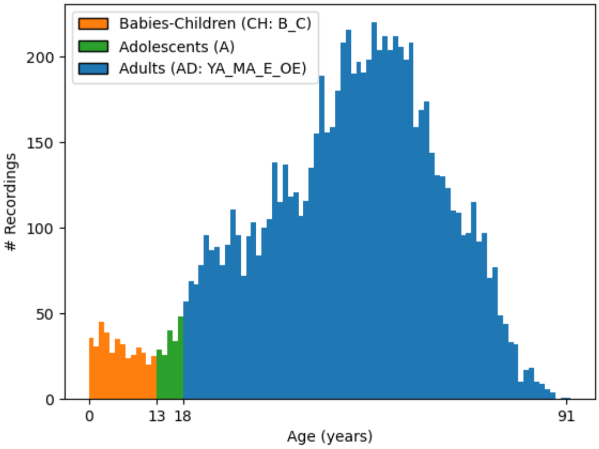

Supplementary Figure 2 Age distribution of the BSDB dataset (n=8869) in the three age groups by AASM [1]. CH: Babies-Children (0-12 y); A: Adolescents (13-18 y); AD: Adults ($\geq$ 19 y).

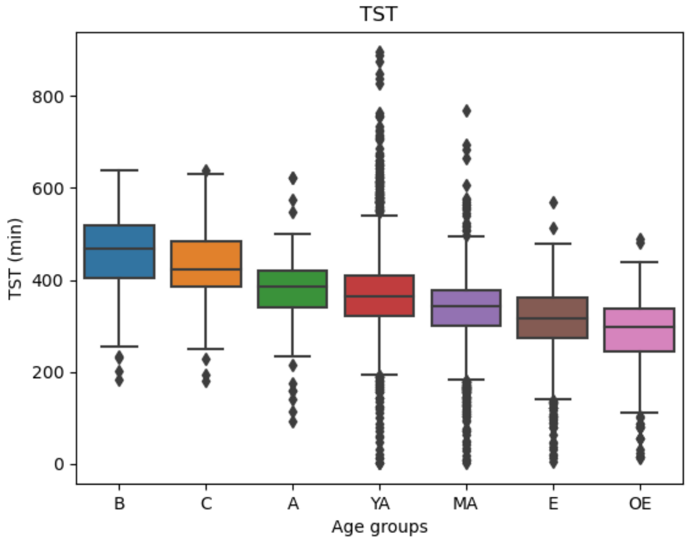

Supplementary Figure 3 Boxplots of the BSDB dataset on the Total Sleep Time (TST) for each of the seven BSDB (G=7) age groups (n=8863). Boxplot shows the minima and maxima values, lower and upper quartiles, and the medians. B: Babies; C: Children; A: Adolescents; YA: Young Adults; MA: Middle Aged Adults; E: Elderly; OE: Old Elderly.

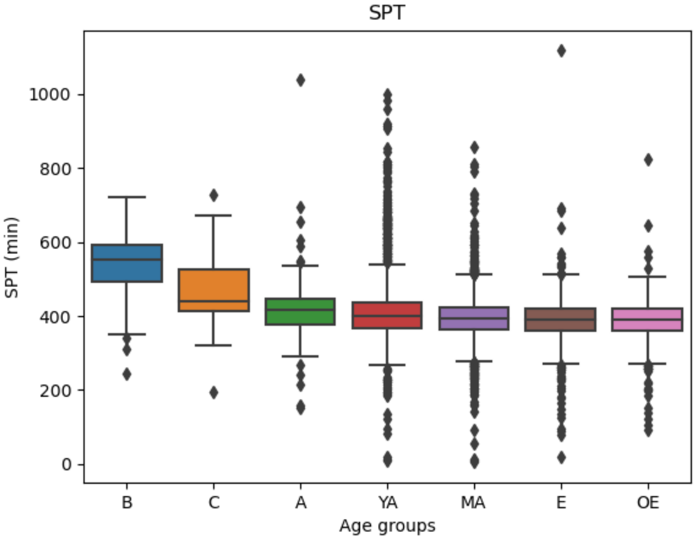

Supplementary Figure 4 Boxplots of the BSDB dataset on the Sleep Period Time (SPT) for each of the seven (G=7) age groups (n=8863). Boxplot shows the minima and maxima values, lower and upper quartiles, and the medians. B: Babies; C: Children; A: Adolescents; YA: Young Adults; MA: Middle Aged Adults; E: Elderly; OE: Old Elderly.

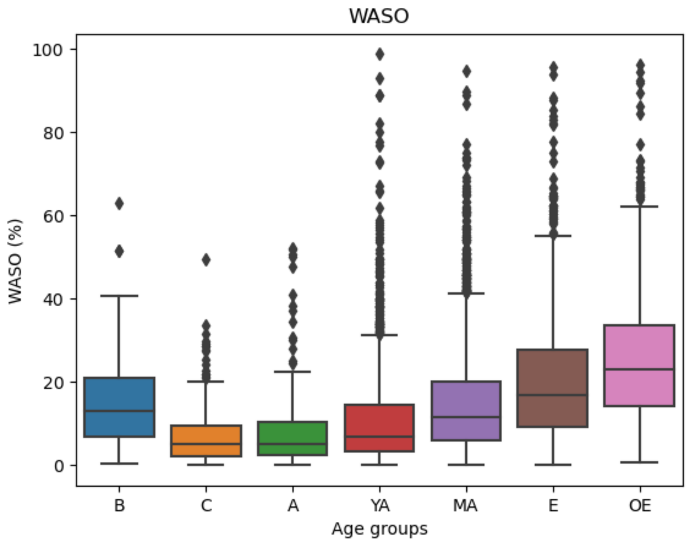

Supplementary Figure 5 Boxplots of the BSDB dataset on the Wake After Sleep Onset (WASO) for each of the seven (G=7) age groups (n=8863). Boxplot shows the minima and maxima values, lower and upper quartiles, and the medians. B: Babies; C: Children; A: Adolescents; YA: Young Adults; MA: Middle Aged Adults; E: Elderly; OE: Old Elderly.

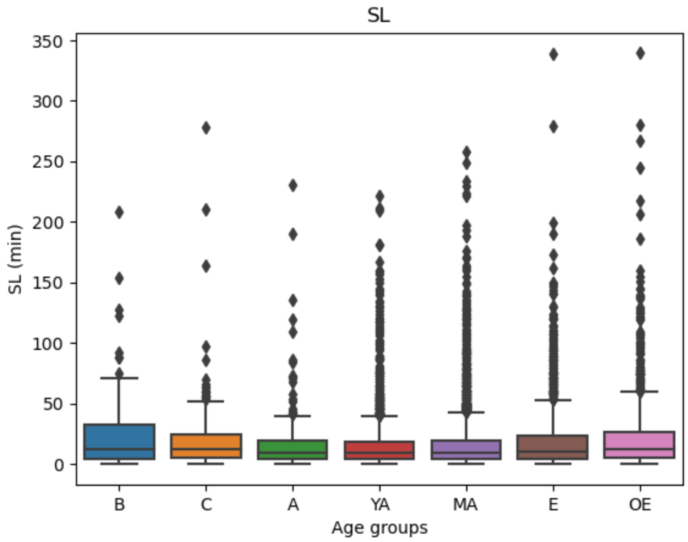

Supplementary Figure 6 Boxplots of the BSDB dataset on the Sleep Latency (SL) for each of the seven (G=7) age groups (n=8863). Boxplot shows the minima and maxima values, lower and upper quartiles, and the medians. B: Babies; C: Children; A: Adolescents; YA: Young Adults; MA: Middle Aged Adults; E: Elderly; OE: Old Elderly.

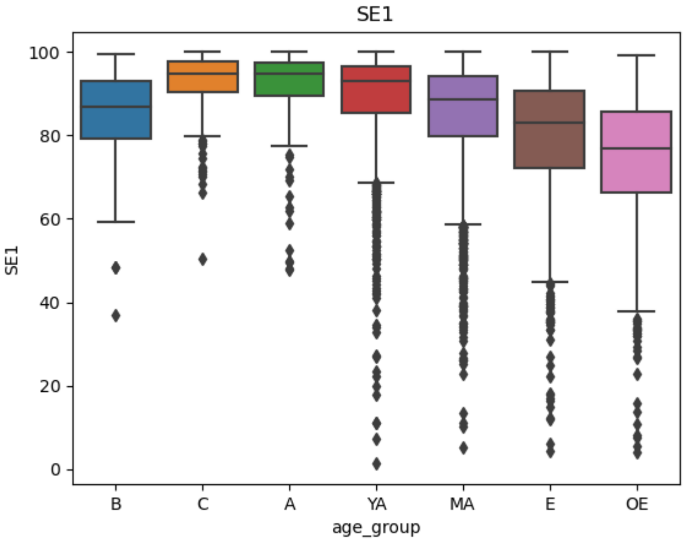

Supplementary Figure 7 Boxplots of the BSDB dataset on the Sleep Efficiency (SE) for each of the seven (G=7) age groups (n=8863). Boxplot shows the minima and maxima values, lower and upper quartiles, and the medians. B: Babies; C: Children; A: Adolescents; YA: Young Adults; MA: Middle Aged Adults; E: Elderly; OE: Old Elderly.

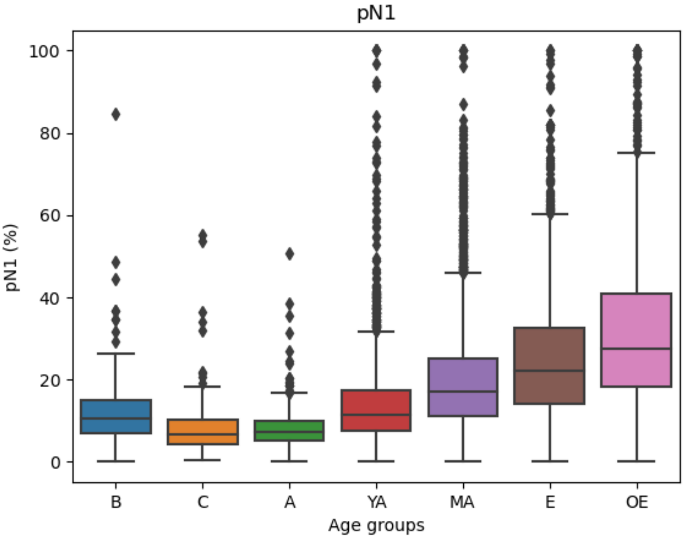

Supplementary Figure 8 Boxplots of the BSDB dataset on the Percentage of N1 stage (pN1) for each of the seven (G=7) age groups (n=8863). Boxplot shows the minima and maxima values, lower and upper quartiles, and the medians. B: Babies; C: Children; A: Adolescents; YA: Young Adults; MA: Middle Aged Adults; E: Elderly; OE: Old Elderly.

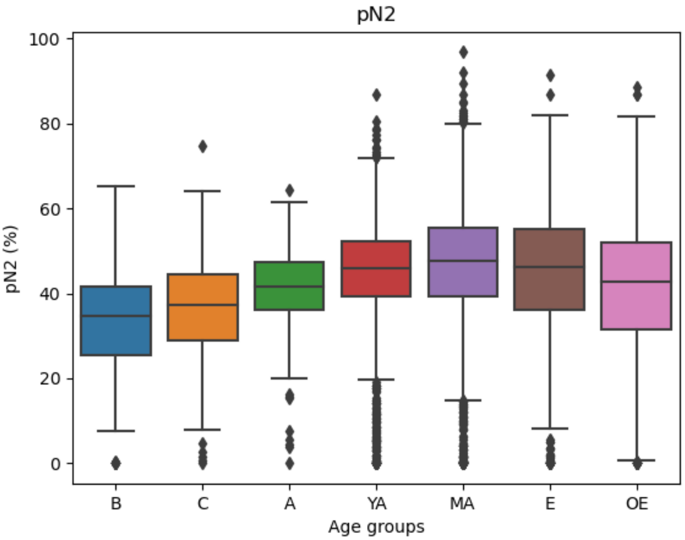

Supplementary Figure 9 Boxplots of the BSDB dataset on the Percentage of N2 stage (pN2) for each of the seven (G=7) age groups (n=8863). Boxplot shows the minima and maxima values, lower and upper quartiles, and the medians. B: Babies; C: Children; A: Adolescents; YA: Young Adults; MA: Middle Aged Adults; E: Elderly; OE: Old Elderly.

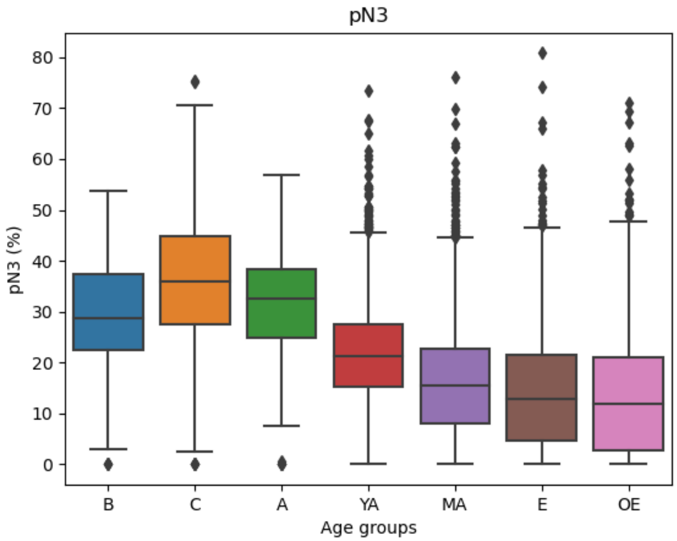

Supplementary Figure 10 Boxplots of the BSDB dataset on the Percentage of N3 stage (pN3) for each of the seven (G=7) age groups (n=8863). Boxplot shows the minima and maxima values, lower and upper quartiles, and the medians. B: Babies; C: Children; A: Adolescents; YA: Young Adults; MA: Middle Aged Adults; E: Elderly; OE: Old Elderly.

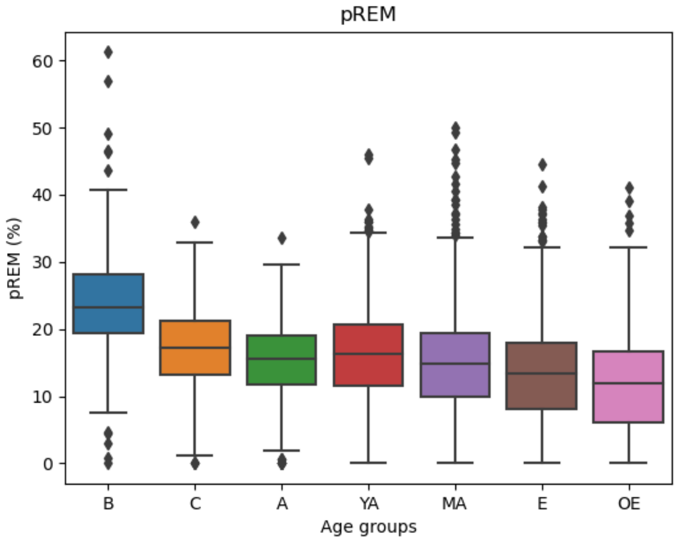

Supplementary Figure 11 Boxplots of the BSDB dataset on the Percentage of REM stage (pREM) for each of the seven (G=7) age groups (n=8863). Boxplot shows the minima and maxima values, lower and upper quartiles, and the medians. B: Babies; C: Children; A: Adolescents; YA: Young Adults; MA: Middle Aged Adults; E: Elderly; OE: Old Elderly.

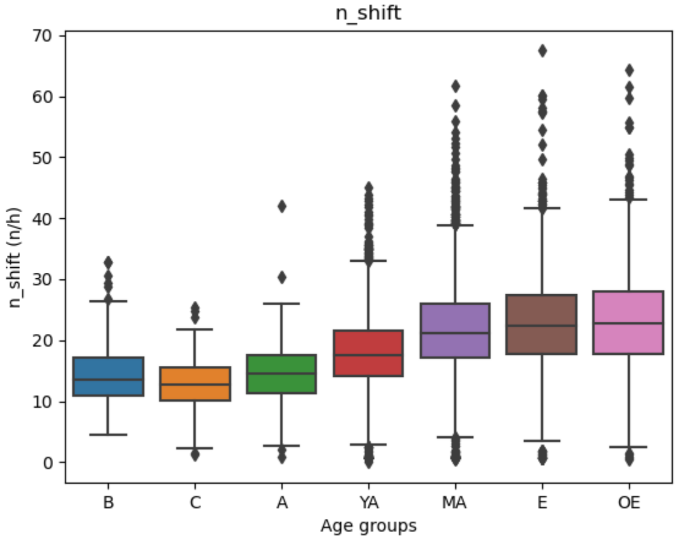

Supplementary Figure 12 Boxplots of the BSDB dataset on the Number of stage shifts per hour (n_shift) for each of the seven (G=7) age groups (n=8863). Boxplot shows the minima and maxima values, lower and upper quartiles, and the medians. B: Babies; C: Children; A: Adolescents; YA: Young Adults; MA: Middle Aged Adults; E: Elderly; OE: Old Elderly.

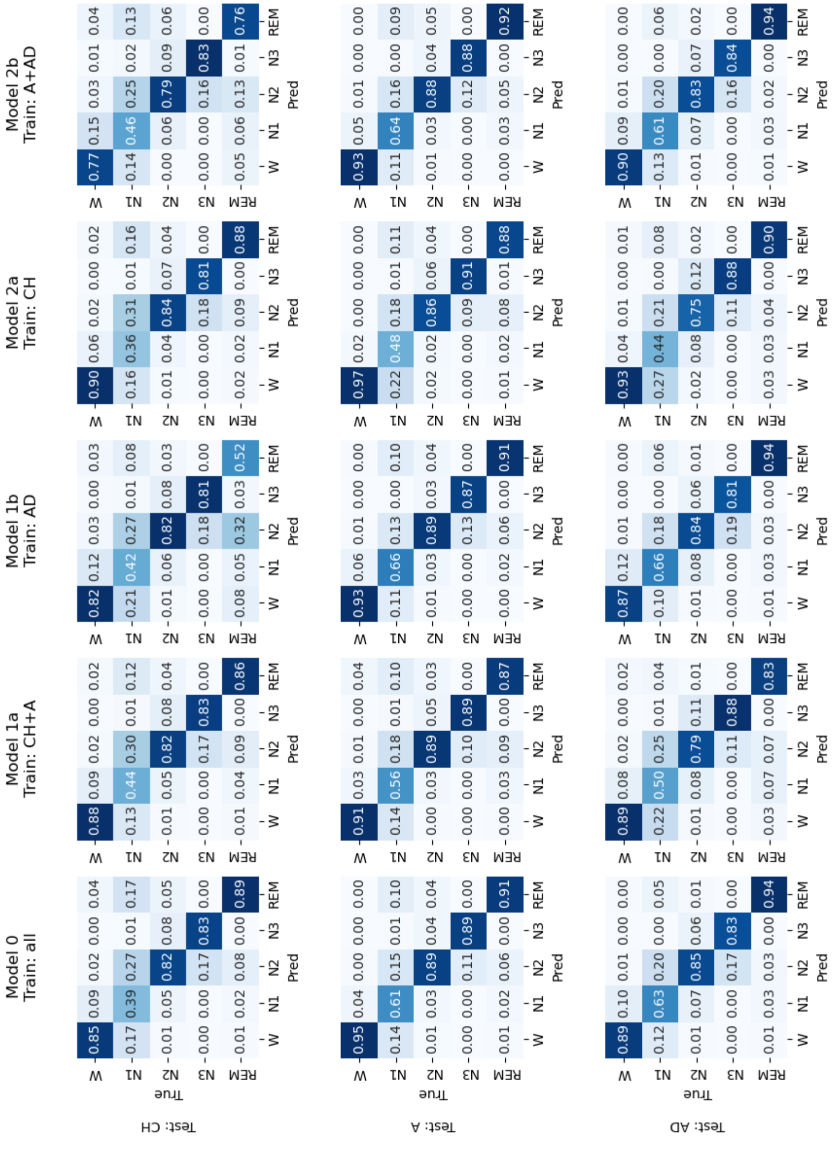

Supplementary Figure 13 ***(iii)*** **Performance of U-Sleep-v1 on five different models, one for each column.** Model 0: fine-tuned on the training set of all the BSDB groups; Model 1a: fine-tuned on the training set of $G_{1a} = \{CH + A\}$; Model 1b: fine-tuned on the training set of $G_{1b} = \{AD\}$; Model 2a: fine-tuned on the training set of $G_{2a} = \{CH\}$; Model 2b: fine-tuned on the training set of $G_{2b} = \{A + AD\}$. All the fine-tuned models are evaluated on the test set of three groups, one for each row: CH, A and AD. We report the confusion matrix for each model and test set. The data split is reported in Supplementary Table 8.
CH: Babies/Children $(B + C)$, A: Adolescents and AD: Adults $(YA + MA + E + OE)$.
B: Babies; C: Children; A: Adolescents; YA: Young Adults; MA: Middle Aged Adults; E: Elderly; OE: Old Elderly.

List of Supplementary Tables

Supplementary Table 1 Age groups by [19] overview with demographic statistics (n=8869). B: Babies; C: Children; A: Adolescents; YA: Young Adults; MA: Middle Aged Adults; E: Elderly; OE: Old Elderly. * The percentage sex % (F/M) is computed on a percentage (in the range of 99.4% - 99.5%) of the total recordings for each age group, given the the lack of availability of the gender information.

Age groups	Recordings	Age range (years)	Age (years)	Sex % (F/M)*
B	151	0-3	1.6 ± 1.1	44/56
C	246	4-12	7.8 ± 2.6	43/57
A	177	13-17	15.3 ± 1.4	41/59
YA	2106	18-39	29.7 ± 6.3	42/58
MA	3655	40-59	50.4 ± 5.5	31/69
E	1546	60-69	64.0 ± 2.8	30/70
OE	988	70-91	75.1 ± 4.1	30/70

Supplementary Table 2 Mean and standard deviation ($\mu \pm \sigma$) of the ten sleep parameters for the seven age groups by [19] (n=8863). TST: Total Sleep Time, SPT: Sleep Period Time, WASO: Wake After Sleep Onset, SL: Sleep Latency, SE: Sleep Efficiency, n_shift: Number of stage shifts per hour, pN1: Percentage of N1 stage, pN2: Percentage of N2 stage, pN3: Percentage of N3 stage, and pREM: Percentage of REM stage. B: Babies; C: Children; A: Adolescents; YA: Young Adults; MA: Middle Aged Adults; E: Elderly; OE: Old Elderly.

Age groups	TST (min)	SPT (min)	WASO (%)	SL (min)	SE	n shift (n/h)	pN1 (%)	pN2 (%)	pN3 (%)	pREM (%)
all	340.2±83.7	402.0±71.4	15.7±13.6	18.1±25.1	84.3±13.6	20.8±7.8	20.2±15.1	44.6±14.2	17.9±12.3	14.5±7.6
B	452.0±85.8	539.6±88.5	15.8±11.4	23.5±30.4	84.2±11.4	14.7±5.4	13.0±10.0	32.6±14.0	29.4±10.9	23.9±9.3
C	430.2±73.3	465.9±75.9	7.5±7.4	20.3±28.3	92.5±7.4	12.9±4.1	8.0±6.6	36.5±11.6	36.5±13.4	17.0±6.9
A	377.8±79.3	415.2±85.0	9.0±10.6	18.7±30.1	91.0±10.6	14.7±5.2	8.9±6.7	41.0±11.1	32.1±10.9	15.1±6.3
YA	371.1±93.9	414.9±90.8	10.9±11.4	16.1±22.4	89.1±11.4	18.1±6.5	14.0±10.9	44.4±13.3	21.4±10.9	15.8±7.3
MA	336.3±66.8	393.7±55.4	14.7±12.1	16.7±22.8	85.3±12.1	21.8±7.4	20.1±13.3	46.5±13.8	16.1±10.9	14.5±7.3
E	311.4±72.6	389.6±56.8	20.3±14.7	20.0±26.5	79.7±14.7	22.8±8.1	25.3±15.9	45.1±14.6	14.5±11.9	13.0±7.5
OE	287.8±73.4	385.6±53.2	25.7±15.7	22.6±32.0	74.3±15.7	23.4±8.3	31.9±19.3	41.5±15.8	13.8±12.2	11.6±7.4

Supplementary Table 3 Age groups by AASM [1] overview with demographic statistics (n=8869). *CH*: Babies/Children ($B + C$), *A*: Adolescents and *AD*: Adults ($YA + MA + E + OE$). * The percentage sex % (F/M) is computed on a percentage (in the range of 99.4% - 99.5%) of the total recordings for each age group, given the the lack of availability of the gender information.

Age groups	Recordings	Age range (years)	Age (years)	Sex % (F/M)*
CH	397	0-12	5.4 ± 3.7	43/57
A	177	13-17	15.3 ± 1.4	41/59
AD	8295	18-91	50.6 ± 15.6	34/66

Supplementary Table 4 Performance of U-Sleep-v1 fine-tuned on $\{CH, A, AD\}$. We report the F1-score (%F1), specifically the mean value and the standard deviation ($\mu \pm \sigma$) computed across the recordings in the test set of $\{CH, A, AD\}$ (data split in Supplementary Table 8). Best shown in bold.

Age groups	Exp.(1a) {CH+A}	Exp. (1b) {AD}	Exp. (2a) {CH}	Exp. (2b) {A AD}
CH	**75.3 ± 8.0**	68.2 ± 12.4	**75.4 ± 7.9**	71.8 ± 10.2
A	76.5 ± 19.1	**82.9 ± 13.7**	78.4 ± 18.1	**82.8 ± 13.6**
AD	72.1 ± 13.0	**77.7 ± 10.9**	72.2 ± 13.3	**77.5 ± 10.8**

Supplementary Table 5 Performance of U-Sleep-v1 with and without label smoothing. Performance of U-Sleep-v1 pre-trained on the OA datasets, and evaluated on all the test set (data split in Supplementary Table 7) of the OA datasets (avg OA) with and without (*i.e.*, U-Sleep-v1 pre-trained in *(ii)*) label smoothing. We report the weighted F1-score (%wF1) and the F1-score (%F1) referred to the epochs kept after the $\mu_{\max}$ query selection procedure (q% threshold value fixed to 5%). We also report the percentage of misclassified epochs among the rejected with query (%miscl.). Specifically, we report the mean value and the standard deviation ($\mu \pm \sigma$) computed across the recordings.

	w/o label smoothing	*w/* label smoothing
%F1	78.1 ± 11.2	72.5 ± 12.4
%wF1	87.4 ± 7.3	82.5 ± 9.8
%miscl.	51.1 ± 9.5	53.7 ± 10.6

Supplementary Table 6 Atypical and/or randomly ordered channel derivations. U-Sleep channel extraction for each open database: (U-Sleep-v0) atypical and/or randomly ordered channel derivations are extracted from the available channels; (U-Sleep-v1) correctly ordered channel derivations are extracted from the available channels, *i.e.*, expected clinical derivations meant to be extracted in [8].

Datasets	Channel type	U-Sleep-v0	U-Sleep-v1
ABC	EEG	F3-F4, O1-C3, C4-F4, E2-C3, O2-F4, M1-C3	F3-M2, F4-M1, C3-M2, C4-M1, O1-M2, O2-M1
	EOG	M2-F4, E1-C3	E1-M2, E2-M1
CCSHS	EEG	LOC-C4, M2-ROC	C3-A2, C4-A1
	EOG	M1-C4, C3-ROC	LOC-A2, ROC-A1
CFS	EEG	A2-A1, C3-C4	C3-A2, C4-A1
	EOG	ROC-A1, LOC-C4	LOC-A2, ROC-A1
CHAT*	EEG	M2-E1, F4-T4, C3-E1, E2-T4, C4-E1, T3-T4, M1-E1, O2-T4	F3-M2, F4-M1, C3-M2, C4-M1, T3-M2, T4-M1, O1-M2, O2-M1
	EOG	O1-E1, F3-T4	E1-M2, E2-M1
DCSM	EEG	F3-M2, F4-M1, C3-M2, C4-M1, O1-M2, O2-M1	F3-M2, F4-M1, C3-M2, C4-M1, O1-M2, O2-M1
	EOG	E1-M2, E2-M1	E1-M2, E2-M1
HPAP**	EEG	-, -, -, -, -, -	F3-M2, F4-M1, C3-M2, C4-M1, O1-M2, O2-M1
	EOG	-, -	E1-M2, E2-M1
MESA	EEG	E2-Fpz, C4-M1, E1-Fpz	Fpz-Cz, Cz-Oz, C4-M1
	EOG	Fz-Cz, Cz-Oz	E1-Fpz, E2-Fpz
MROS	EEG	E1-C4, M1-C3	C3-M2, C4-M1
	EOG	M2-C4, E2-C3	LOC-M2, ROC-M1
PHYS	EEG	F3-M2, F4-M1, C3-M2, C4-M1, O1-M2, O2-M1	F3-M2, F4-M1, C3-M2, C4-M1, O1-M2, O2-M1
	EOG	E1-M2	E1-M2
SEDF-SC	EEG	Pz-Oz, Fpz-Cz	Fpz-Cz, Pz-Oz
	EOG	EOG horizontal	EOG horizontal
SEDF-ST	EEG	Pz-Oz, Fpz-Cz	Fpz-Cz, Pz-Oz
	EOG	EOG horizontal	EOG horizontal
SHHS	EEG	C4-A1, C3-A2	C4-A1, C3-A2
	EOG	EOGL-PG1, EOGR-PG1	EOGL-PG1, EOGR-PG1
SOF	EEG	LOC-A2, A1-C4	C3-A2, C4-A1
	EOG	C3-A2, ROC-C4	LOC-A2, ROC-A1

*The CHAT dataset has recordings where we may find a different order of EEG and EOG sensors for different edf files. Consequently, in the U-Sleep version where they were erroneously extracting atypical and/or randomly ordered channel derivations (U-Sleep-v0), we can generate multiple combinations of incorrect derivations. In Table we report the most frequent incorrect EEG and EOG derivations.

**The HPAP dataset has recordings where we can find a different order of EEG and EOG sensors for each edf file, resulting in different combinations of incorrect derivations for each recording. For that reason, we preferred not to report the incorrect and completely random combinations of derivations between the different recordings.

Supplementary Table 7 Data split on the OA datasets. We report the total number of recordings, and the number of recordings used to train, validate and test the U-Sleep architecture in the experiments *(i)* and *(ii)* for each open access dataset.

Datasets	Recordings	Train	Valid	Test
ABC	132	93	15	24
CCSHS	515	387	50	78
CFS	730	531	95	104
CHAT	1638	1438	70	130
DCSM	255	190	26	39
HPAP	238	178	24	36
MESA	2056	1906	50	100
MROS	3926	3728	69	129
PHYS	994	844	50	100
SEDF-SC	153	115	15	23
SEDF-ST	44	30	6	8
SHHS	8444	8226	77	141
SOF	453	339	46	68

Supplementary Table 8 Data split on the BSDB dataset. We report the total number of recordings, and the number of recordings used to train, validate and test the U-Sleep architecture in the experiments *(i)*, *(ii)* and *(iii)* for each age group of the BSDB dataset.

Experiments	Age groups	Recordings	Train	Valid	Test
(i) (ii)	-	8884	6658	882	1344
(iii)	B	151	111	14	26
	C	246	185	26	35
	A	177	132	17	28
	YA	2066	1902	58	106
	MA	3636	3482	51	103
	E	1539	1378	55	106
	OE	988	829	53	106

Springer Nature 2021 LaTeX template

Supplementary Table 9 ***(i)* Clinically non-recommended channel derivations (per class F1-score).** Performance of U-Sleep-v0 and U-Sleep-v1, pre-trained on the OA datasets, and evaluated on the test set of the BSDB dataset (data split in Supplementary Table 8), and on the whole $BSDB_{(100\%)}$ dataset, *i.e.*, both direct transfer (DT) on BSDB. We report the per class F1-score computed across the recordings.

	Datasets	W	N1	N2	N3	REM
v0	BSDB	81.1 ± 14.4	49.3 ± 16.7	79.0 ± 15.1	72.3 ± 25.2	83.8 ± 19.9
v1	BSDB	82.3 ± 14.4	47.2 ± 16.5	79.7 ± 14.5	71.0 ± 26.2	85.3 ± 18.5
v0	$BSDB_{(100\%)}$	81.5 ± 14.4	50.1 ± 16.8	79.6 ± 15.0	72.9 ± 24.8	84.1 ± 19.0
v1	$BSDB_{(100\%)}$	82.6 ± 14.3	48.2 ± 16.9	80.2 ± 14.6	71.4 ± 26.1	85.5 ± 17.5

Supplementary Table 10 ***(i)* Clinically non-recommended channel derivations (weighted F1-score and Cohen's Kappa).** Performance of U-Sleep-v0 and U-Sleep-v1, pre-trained on the OA datasets, and evaluated on the test set of the BSDB dataset (data split in Supplementary Table 8), and on the whole $BSDB_{(100\%)}$ dataset, *i.e.*, both direct transfer (DT) on BSDB. We report the weighted F1-score (%wF1), specifically the mean value and the standard deviation ($\mu \pm \sigma$) computed across the recordings. We also report the overall Cohen's Kappa coefficient (k) computed across all the recordings.

%wF1		
Datasets	U-Sleep-v0	U-Sleep-v1
BSDB	77.8 ± 10.9	77.9 ± 10.8
$BSDB_{(100\%)}$	78.2 ± 11.1	78.3 ± 11.2
k		
Datasets	U-Sleep-v0	U-Sleep-v1
BSDB	0.72	0.72
$BSDB_{(100\%)}$	0.72	0.73

Supplementary Table 11 ***(ii)*** **Generalizability on different data centers with a heterogeneous dataset (weighted F1-score and Cohen's Kappa).** Performance of U-Sleep-v1, pre-trained on the OA datasets, and evaluated on all the test set of the OA datasets (data split in Supplementary Table 7) and on the test set of the BSDB dataset (data split in Supplementary Table 8). We also report the performance of U-Sleep-v1 trained from scratch (S) or fine-tuned (FT) on the BSDB dataset, and evaluated on all the test set of all the available datasets. We report the weighted F1-score (%wF1), specifically the mean value and the standard deviation ($\mu \pm \sigma$) computed across the recordings. We also report the overall Cohen's Kappa coefficient (k) computed across all the recordings.

%wF1			
Datasets	U-Sleep-v1	U-Sleep-v1 (S)	U-Sleep-v1 (FT)
ABC	81.3 ± 8.5	78.9 ± 10.1	76.5 ± 10.1
CCSHS	90.3 ± 4.6	85.3 ± 6.3	85.4 ± 5.9
CFS	87.8 ± 6.6	82.2 ± 7.9	82.8 ± 7.2
CHAT	86.4 ± 4.8	79.3 ± 6.7	76.5 ± 7.5
DCSM	90.5 ± 4.4	81 ± 8.9	79.1 ± 9.7
HPAP	80.6 ± 7.5	75.8 ± 9.6	74.5 ± 11.7
MESA	84.2 ± 7.2	79.1 ± 12.9	79.6 ± 9.3
MROS	85.3 ± 7.0	75.5 ± 10.5	77.4 ± 9.8
PHYS	80.5 ± 8.6	79.2 ± 8.9	79.2 ± 9.0
SEDF-SC	86.7 ± 5.5	85.1 ± 5.6	86.6 ± 5.3
SEDF-ST	83.5 ± 4.5	73.6 ± 8.3	74.9 ± 6.4
SHHS	86.6 ± 6.3	81.6 ± 7.1	83.5 ± 6.5
SOF	85.6 ± 6.5	75.6 ± 10.9	78.4 ± 9.9
avg OA	85.7 ± 7.1	79.7 ± 9.4	80.0 ± 9.0
BSDB	77.9 ± 10.8	82.2 ± 9.4	82.0 ± 9.4
k			
Datasets	U-Sleep-v1	U-Sleep-v1 (S)	U-Sleep-v1 (FT)
ABC	0.76	0.72	0.68
CCSHS	0.86	0.78	0.78
CFS	0.83	0.72	0.73
CHAT	0.82	0.72	0.69
DCSM	0.86	0.69	0.66
HPAP	0.74	0.67	0.65
MESA	0.78	0.71	0.70
MROS	0.79	0.62	0.64
PHYS	0.74	0.72	0.72
SEDF-SC	0.82	0.79	0.81
SEDF-ST	0.78	0.64	0.66
SHHS	0.82	0.73	0.76
SOF	0.81	0.64	0.67
avg OA	0.81	0.71	0.71
BSDB	0.72	0.77	0.77

Supplementary Table 12 ***(iii)*** **Training conditioned by age (weighted F1-score and Cohen's Kappa).** Performance of U-Sleep-v1 on a single model fine-tuned on all the training set of the seven BSDB groups (FT); on seven/two models fine-tuned on the independent training set of each group (FT-I) with $G = 7$ and $G = 2$ respectively; and on a single model fine-tuned on all the training set of the seven/two BSDB groups conditioned (FT-SaBN) by $G = 7$ and by $G = 2$ groups respectively. All the fine-tuned models are evaluated on the associated test set of each group (data split in Supplementary Table 8). We report the weighted F1-score (%wF1), specifically the mean value and the standard deviation ($\mu \pm \sigma$) computed across the recordings. We also report the overall Cohen's Kappa coefficient (k) computed across all the recordings. B: Babies (0-3 years); C: Children (4-12 years); A: Adolescents (13-18 years); YA: Young Adults (19-39 years); MA: Middle Aged Adults (40-59 years); E: Elderly (60-69 years); OE: Old Elderly ($\geq$ 70 years). When $G = 2$ we have the following two groups $G_1 = \{B + C\}$, $G_2 = \{A + YA + MA + E + OE\}$.

%wF1					
Age groups	FT-G1	FT-I-G7	FT-I-G2	FT-SaBN-G7	FT-SaBN-G2
B	79.2 ± 7.4	78.7 ± 7.2 ^{G_1}	77.6 ± 8.5 ^{G_1}	79.5 ± 6.7	77.2 ± 7.9
C	80.8 ± 9.5	80.7 ± 8.9 ^{G_2}	81.1 ± 7.7 ^{G_1}	81.5 ± 8.0	81.4 ± 7.9
A	87.3 ± 10.8	86.0 ± 13.4 ^{G_3}	87.0 ± 10.7 ^{G_2}	87.0 ± 10.8	86.2 ± 11.3
YA	85.9 ± 9.3	85.6 ± 9.4 ^{G_4}	85.4 ± 9.5 ^{G_2}	85.5 ± 9.4	84.6 ± 9.8
MA	84.1 ± 6.2	83.5 ± 6.6 ^{G_5}	83.4 ± 6.4 ^{G_2}	83.5 ± 6.6	82.9 ± 6.9
E	80.5 ± 8.3	79.4 ± 9.0 ^{G_6}	79.6 ± 8.9 ^{G_2}	80.0 ± 8.2	79.0 ± 9.4
OE	79.8 ± 9.6	78.9 ± 9.4 ^{G_7}	79.1 ± 9.7 ^{G_2}	79.4 ± 9.5	78.8 ± 9.4
avg	82.5 ± 9.0	81.8 ± 9.4	81.9 ± 9.2	82.2 ± 8.9	81.4 ± 9.34
k					
Age groups	FT-G1	FT-I-G7	FT-I-G2	FT-SaBN-G7	FT-SaBN-G2
B	0.73	0.73 ^{G_1}	0.73 ^{G_1}	0.71	0.70
C	0.73	0.73 ^{G_2}	0.74 ^{G_1}	0.74	0.74
A	0.83	0.82 ^{G_3}	0.83 ^{G_2}	0.83	0.82
YA	0.82	0.81 ^{G_4}	0.82 ^{G_2}	0.81	0.80
MA	0.78	0.78 ^{G_5}	0.78 ^{G_2}	0.78	0.77
E	0.74	0.73 ^{G_6}	0.73 ^{G_2}	0.73	0.72
OE	0.73	0.72 ^{G_7}	0.73 ^{G_2}	0.72	0.72
avg	0.78	0.77	0.77	0.77	0.76

References

[1] Berry, R.B., Budhiraja, R., Gottlieb, D.J., Gozal, D., Iber, C., Kapur, V.K., Marcus, C.L., Mehra, R., Parthasarathy, S., Quan, S.F., *et al.*: Rules for scoring respiratory events in sleep: update of the 2007 aasm manual for the scoring of sleep and associated events: deliberations of the sleep apnea definitions task force of the american academy of sleep medicine. Journal of clinical sleep medicine **8**(5), 597–619 (2012)

[2] Zhang, G.-Q., Cui, L., Mueller, R., Tao, S., Kim, M., Rueschman, M., Mariani, S., Mobley, D., Redline, S.: The national sleep research resource: towards a sleep data commons. Journal of the American Medical Informatics Association **25**(10), 1351–1358 (2018)

[3] Bakker, J.P., Tavakkoli, A., Rueschman, M., Wang, W., Andrews, R., Malhotra, A., Owens, R.L., Anand, A., Dudley, K.A., Patel, S.R.: Gastric banding surgery versus continuous positive airway pressure for obstructive sleep apnea: a randomized controlled trial. American journal of respiratory and critical care medicine **197**(8), 1080–1083 (2018)

[4] Rosen, C.L., Larkin, E.K., Kirchner, H.L., Emancipator, J.L., Bivins, S.F., Surovec, S.A., Martin, R.J., Redline, S.: Prevalence and risk factors for sleep-disordered breathing in 8-to 11-year-old children: association with race and prematurity. The Journal of pediatrics **142**(4), 383–389 (2003)

[5] Redline, S., Tishler, P.V., Tosteson, T.D., Williamson, J., Kump, K., Browner, I., Ferrette, V., Krejci, P.: The familial aggregation of obstructive sleep apnea. American journal of respiratory and critical care medicine **151**(3), 682–687 (1995)

[6] Marcus, C.L., Moore, R.H., Rosen, C.L., Giordani, B., Garetz, S.L., Taylor, H.G., Mitchell, R.B., Amin, R., Katz, E.S., Arens, R., *et al.*: A randomized trial of adenotonsillectomy for childhood sleep apnea. N Engl J Med **368**, 2366–2376 (2013)

[7] Redline, S., Amin, R., Beebe, D., Chervin, R.D., Garetz, S.L., Giordani, B., Marcus, C.L., Moore, R.H., Rosen, C.L., Arens, R., *et al.*: The childhood adenotonsillectomy trial (chat): rationale, design, and challenges of a randomized controlled trial evaluating a standard surgical procedure in a pediatric population. Sleep **34**(11), 1509–1517 (2011)

[8] Perslev, M., Darkner, S., Kempfner, L., Nikolic, M., Jennum, P.J., Igel, C.: U-sleep: resilient high-frequency sleep staging. NPJ digital medicine **4**(1), 1–12 (2021)

[9] Rosen, C.L., Auckley, D., Benca, R., Foldvary-Schaefer, N., Iber, C., Kapur, V., Rueschman, M., Zee, P., Redline, S.: A multisite randomized

trial of portable sleep studies and positive airway pressure autotitration versus laboratory-based polysomnography for the diagnosis and treatment of obstructive sleep apnea: the homepap study. Sleep **35**(6), 757–767 (2012)

[10] Chen, X., Wang, R., Zee, P., Lutsey, P.L., Javaheri, S., Alcántara, C., Jackson, C.L., Williams, M.A., Redline, S.: Racial/ethnic differences in sleep disturbances: the multi-ethnic study of atherosclerosis (mesa). Sleep **38**(6), 877–888 (2015)

[11] Blackwell, T., Yaffe, K., Ancoli-Israel, S., Redline, S., Ensrud, K.E., Stefanick, M.L., Laffan, A., Stone, K.L., in Men Study Group, O.F.: Associations between sleep architecture and sleep-disordered breathing and cognition in older community-dwelling men: the osteoporotic fractures in men sleep study. Journal of the American Geriatrics Society **59**(12), 2217–2225 (2011)

[12] Relationships between sleep stages and changes in cognitive function in older men: the mros sleep study. Sleep **38**(3), 411–421 (2015)

[13] Goldberger, A.L., Amaral, L.A., Glass, L., Hausdorff, J.M., Ivanov, P.C., Mark, R.G., Mietus, J.E., Moody, G.B., Peng, C.-K., Stanley, H.E.: Physiobank, physiotoolkit, and physionet: components of a new research resource for complex physiologic signals. circulation **101**(23), 215–220 (2000)

[14] Ghassemi, M.M., Moody, B.E., Lehman, L.-W.H., Song, C., Li, Q., Sun, H., Mark, R.G., Westover, M.B., Clifford, G.D.: You snooze, you win: the physionet/computing in cardiology challenge 2018. In: 2018 Computing in Cardiology Conference (CinC), vol. 45, pp. 1–4 (2018). IEEE

[15] Kemp, B., Zwinderman, A.H., Tuk, B., Kamphuisen, H.A., Oberye, J.J.: Analysis of a sleep-dependent neuronal feedback loop: the slow-wave microcontinuity of the eeg. IEEE Transactions on Biomedical Engineering **47**(9), 1185–1194 (2000)

[16] Quan, S.F., Howard, B.V., Iber, C., Kiley, J.P., Nieto, F.J., O'Connor, G.T., Rapoport, D.M., Redline, S., Robbins, J., Samet, J.M., *et al.*: The sleep heart health study: design, rationale, and methods. Sleep **20**(12), 1077–1085 (1997)

[17] Cummings, S.R., Black, D.M., Nevitt, M.C., Browner, W.S., Cauley, J.A., Genant, H.K., Mascioli, S.R., Scott, J.C., Seeley, D.G., Steiger, P., *et al.*: Appendicular bone density and age predict hip fracture in women. Jama **263**(5), 665–668 (1990)

[18] Spira, A.P., Blackwell, T., Stone, K.L., Redline, S., Cauley, J.A., Ancoli-Israel, S., Yaffe, K.: Sleep-disordered breathing and cognition in older women. Journal of the American Geriatrics Society **56**(1), 45–50 (2008)

[19] Ohayon, M., Carskadon, M., Guilleminault, C., Vitiello, M.: Meta-analysis of quantitative sleep parameters from childhood to old age in healthy individuals: Developing normative sleep values across the human lifespan. Sleep **27**, 1255–73 (2004)

[20] Danker-hopfe, H., Anderer, P., Zeitlhofer, J., Boeck, M., Dorn, H., Gruber, G., Heller, E., Loretz, E., Moser, D., Parapatics, S., *et al.*: Interrater reliability for sleep scoring according to the rechtschaffen & kales and the new aasm standard. Journal of sleep research **18**(1), 74–84 (2009)

[21] Rosenberg, R.S., Van Hout, S.: The american academy of sleep medicine inter-scorer reliability program: sleep stage scoring. Journal of clinical sleep medicine **9**(01), 81–87 (2013)

[22] Younes, M., Raneri, J., Hanly, P.: Staging sleep in polysomnograms: analysis of inter-scorer variability. Journal of Clinical Sleep Medicine **12**(06), 885–894 (2016)

[23] Muto, V., Berthomier, C., Schmidt, C., Vandewalle, G., Jaspar, M., Devillers, J., Chellappa, S., Meyer, C., Phillips, C., Berthomier, P., *et al.*: 0315 inter-and intra-expert variability in sleep scoring: comparison between visual and automatic analysis. Sleep **41**(suppl_1), 121 (2018)

[24] Goodfellow, I., Bengio, Y., Courville, A.: Deep Learning. MIT Press, ??? (2016)

[25] Lukasik, M., Bhojanapalli, S., Menon, A., Kumar, S.: Does label smoothing mitigate label noise? In: International Conference on Machine Learning, pp. 6448–6458 (2020). PMLR

[26] Fiorillo, L., Favaro, P., Faraci, F.D.: Deepsleepnet-lite: A simplified automatic sleep stage scoring model with uncertainty estimates. IEEE Transactions on Neural Systems and Rehabilitation Engineering **29**, 2076–2085 (2021)

[27] Szegedy, C., Vanhoucke, V., Ioffe, S., Shlens, J., Wojna, Z.: Rethinking the inception architecture for computer vision. In: Proceedings of the IEEE Conference on Computer Vision and Pattern Recognition, pp. 2818–2826 (2016)
